# Alternative splicing regulates *FGGY-*derived neoantigen presentation and promotes immune evasion in metabolic-associated hepatocellular carcinoma

**DOI:** 10.1016/j.isci.2026.115999

**Published:** 2026-06-06

**Authors:** Li Na Zhao, Jesper B. Andersen

**Affiliations:** 1 Department of Health and Medical Sciences, Biotech Research and Innovation Centre (BRIC), University of Copenhagen, Copenhagen, Denmark

**Keywords:** Cancer, In silico biology

## Abstract

The elucidation of actionable, tumor-specific neoantigens remains a challenge in cancer immunotherapy. We identify *FGGY*-204, an alternatively spliced isoform of *FGGY*, as a source of tumor-specific neoantigen presentation. *FGGY*-204 expression is elevated in HCC and encodes a cryptic peptide that is derived from the primate-specific exon 12. This neoantigen candidate peptide is repressed in the normal liver parenchyma by a multifaceted regulatory program, where the *FGGY* exon 12 locus is epigenetically silenced by abrogated active chromatin marks and DNA methylation. Exon 12 inclusion is directly suppressed by HNRNPA1, whose silencing promotes *FGGY*-204 biogenesis, revealing a repressive mechanism with potential in therapy. Critically, *FGGY*-204 expression defines immune-cold HCC tumors, inversely correlated with CD8^+^ T cell tumor infiltration and antigen presentation. Its immune association dynamically shifts in response to immunotherapy. *FGGY*-204 presents a neopeptide, a potential immunotherapeutic target, revealing its precise molecular mechanism of control as an entry point to modulate tumor immunogenicity.

## Highlights

• FGGY-204 exon 12 generates a tumor-specific neoantigen in MASLD-related HCC
• Poised chromatin and HNRNPA1 repress FGGY-204 exon 12 inclusion
• SMARCA4-KDM8-CDK9 axis regulates canonical FGGY-201 transcription
• FGGY-204 marks immune-cold HCC and dynamically responds to immunotherapy

## Graphical abstract

**Figure:**
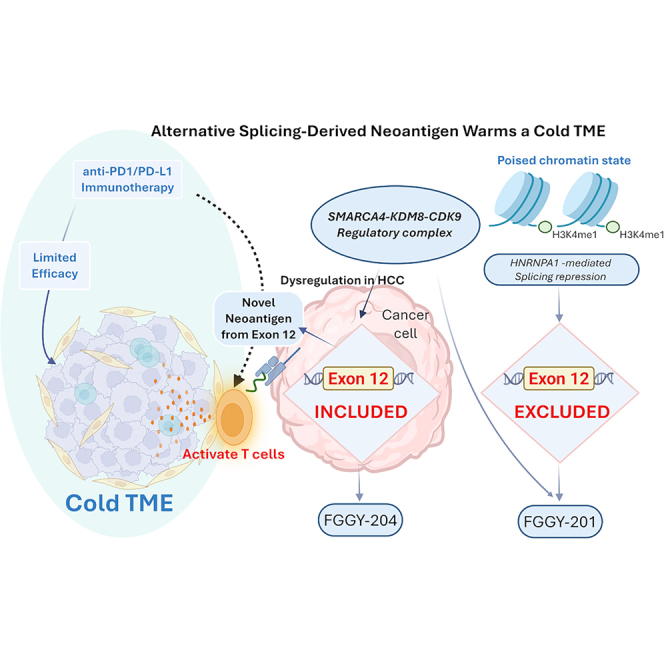

## Introduction

Hepatocellular carcinoma (HCC) driven by metabolic dysfunction-associated steatotic liver disease (MASLD) represents a growing global health burden with limited therapeutic options.^1^ While immune checkpoint inhibitors (ICIs) show promise, the objective response rates (ORRs) remain relatively low (less than 20%),^2^^,^^3^ partly attributable to the lack of targetable neoantigens. Higher ORRs can be achieved with combination therapies. Paradoxically, MASLD-HCC exhibits moderate mutational burden,^4^ nevertheless poor immunogenicity,^1^ suggesting that non-canonical sources of neoantigens may be critical for effective immunotherapy.^5^

Most neoantigen discovery efforts are focused on somatic mutations, gene fusions, and structural variants,^6^^,^^7^^,^^8^^,^^9^^,^^10^^,^^11^^,^^12^ while antigens derived from alternative splicing events, a process generating over 95% of human protein diversity, remain limited.^12^^,^^13^^,^^14^ Splicing-derived neoantigens are particularly appealing in MASLD-HCC, where metabolic rearrangements and chronic inflammation promote widespread isoform dysregulation.^15^^,^^16^^,^^17^^,^^18^ However, the mechanism through which the chromatin state influences splicing decisions and consequently shapes neoantigen presentation in HCC remains largely unknown.

The carbohydrate kinase domain-containing gene FGGY encodes a conserved metabolic enzyme within the carbohydrate kinase family.^19^^,^^20^ While known for its role in sugar metabolism, FGGY undergoes alternative splicing. We identify an FGGY isoform (FGGY-204) associated with splicing-dependent neoantigen generation in MASLD-HCC. Specifically, exon 12, which presents a primate-specific locus, encodes a tumor-enriched neopeptide that is detectable by mass spectrometry (MS). FGGY-204 is epigenetically regulated by a process where priming of histone 3 lysine 4 monomethylation (H3K4me1) poises exon 12 for inclusion in HCC tumors, while the RNA-binding protein HNRNPA1 is repressing it by binding to FGGY intron 11, thereby creating a competitive splicing switch. This isoform switch is modulated by SMARCA4-mediated chromatin accessibility. Through the crosstalk of chromatin modifications and splicing, this regulatory mechanism generates an immunogenic neopeptide that inversely correlates with intratumoral CD8^+^ T cell infiltration, suggesting that FGGY-204 expression is involved in promoting tumor-specific immune evasion.

We demonstrate that coordinated epigenetic regulation and splicing control can generate clinically actionable neoantigens independent of somatic mutations. Specifically, the *FGGY*-204 exon 12 neopeptide, which is absent in the healthy human liver and from murine orthologs, emerges as a compelling onco-vaccine target enriched in MASLD-HCC. This isoform-specific neoantigen exemplifies how chromatin state control and splicing factor competition may establish “ready-to-present” targets in the tumor setting. Importantly, its regulation that involves HNRNPA1 and SMARCA4 control provides further pharmacologic entry points to amplify antigenicity, with possible HNRNPA1 inhibition offering a strategy to enhance neoantigen presentation in combination with immune checkpoint blockade. These findings define a paradigm for leveraging alternative splicing-derived neopeptides to overcome a predominant immunologically cold tumor type in MASLD-HCC.

## Results

### Identification of a tumor-specific FGGY isoform encoding a neopeptide

**Figure 1:**
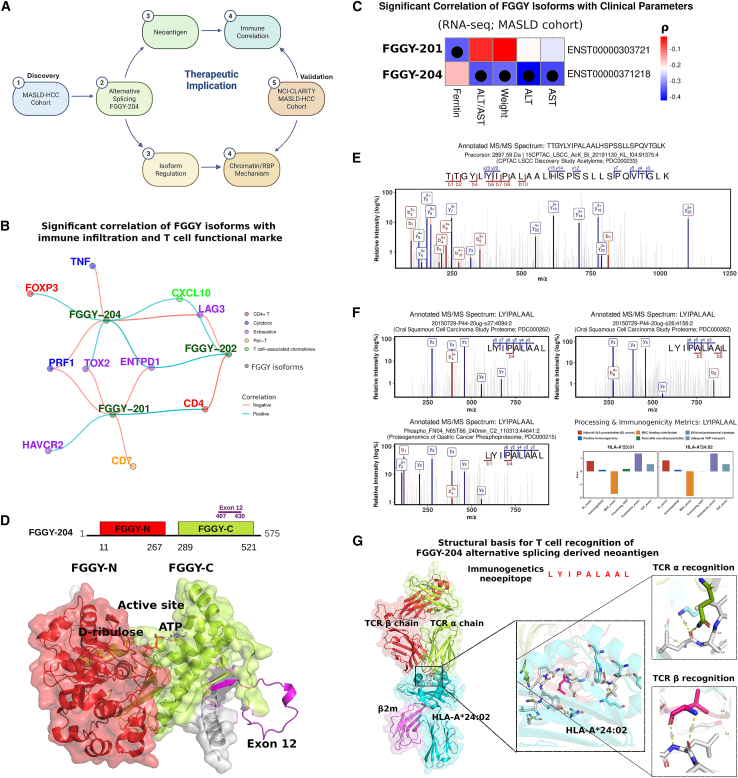
Integrated clinical, metabolic, and single-cell transcriptomic associations highlight FGGY isoform-specific links to liver pathology (A) Study flow. (B) Correlation network between FGGY isoforms and T cell-associated genes. Spearman correlations (absolute r > 0.4, FDR<0.05) were computed between *FGGY* isoform expression and T cell-related transcript expression in the MAFLD-HCC cohort. Nodes represent either FGGY isoforms (dark green) or T cell marker genes (colored by functional category). Edges are colored by correlation type (cyan, positive; red, negative). The network highlights isoform-specific and antagonistic relationships, underscoring distinct roles of FGGY isoforms in shaping T cell-associated programs. (C) Heatmap showing significant Spearman correlations between FGGY isoforms and clinical parameters. Black dots indicate correlations with absolute values ≥ 0.3 and statistically significant (*p* < 0.05), highlighting notable associations of the FGGY-204 isoform with liver injury markers and weight. (D) Modeled structure of FGGY (AlphaFold). The N terminus is shown in red, the C terminus is shown in lime green, and exon 12 is highlighted in purple in cartoon representation. Exon 12 is positioned away from the predicted active site. (E) Representative high-confidence MS/MS spectrum of the FGGY-204-specific peptide TTGYLYIPALAALHSPSSLLSPQVTGLK. The annotated spectrum was obtained from the CPTAC LSCC Discovery Study Acetylome (PDC000233) and corresponds to precursor ion 15CPTAC_LSCC_AcK_BI_20191130_KL_f04:91375:4 (charge 4+). Observed fragment ions are annotated as b-ions (dark red) and y-ions (dark blue), with subscripts denoting fragment position and superscripts indicating charge state. Unmatched peaks are shown in light gray for reference. Peak intensities are plotted on a log scale to highlight low-abundance fragments. This peptide was also confidently identified in multiple additional spectra across datasets, including as doubly and triply charged ions. In addition, four cryptic peptides associated with exon 12 were detected across multiple proteomics datasets. (F) Representative identification of the immunogenetic core epitope LYIPALAAL. Peptide-spectrum matches corresponding to the core epitope LYIPALAAL were detected with high confidence in multiple datasets (PDC000262, PDC000215). Observed spectra consistently mapped to doubly charged precursor ions (m/z ∼1,087.68, charge 2+) with strong fragmentation support. The recurring detection of this core sequence across independent tumor proteome datasets supports its reproducibility and immunogenetic relevance. Processing and immunogenicity predictions for peptide LYIPALAAL indicate efficient proteasomal cleavage, adequate TAP transport, positive immunogenicity, and favorable overall presentation, supporting its potential as a CD8^+^ T cell epitope across HLA-A24:02 and HLA-A23:01. (G) Structural basis for T cell recognition of *FGGY*-204 alternative splicing-derived neoantigen. Homology model of the HLA-A24:02-peptide-TCR complex. The structure was generated using Modeller, with 6EQB and 8SBK as templates. Among 20 models, the one with the lowest objective function was selected. Our immunogenetics epitope (LYIPALAAL), interacting strongly interaction with HLA-A24:02 (cyan). The TCR α-chain is highlighted in red, and the TCR β-chain in lime, illustrating the recognition interface of the TCR with the peptide-MHC complex.

The metabolic enzyme FGGY is encoded by a complex gene locus, which can give rise to multiple isoforms, presenting distinct roles. To systematically investigate FGGY alternative splicing and transcriptional control in HCC, we designed a multi-step workflow (Figure 1A), integrating transcriptomic analyses, isoform-level quantification, and correlation with immune gene expression. Using this approach, we compared FGGY isoform expression in HCC tumors from a well-characterized MASLD cohort (MASLD-HCC) with matched adjacent normal liver tissues.

We identified a tumor-specific pattern of *FGGY* isoform switching. Specifically, the *FGGY*-204 (ENST00000371218) and the related *FGGY*-202 (ENST00000371210) isoforms are significantly upregulated in HCC tumors compared to the matched adjacent normal livers. These data were replicated in independent HCC cohorts (Figures S1A–S1C) representing more than 100 patients with paired tumor/normal data. Contrarily, the canonical *FGGY* transcript (*FGGY*-201; ENST00000303721) is consistently downregulated in HCC tumors (TCGA-LIHC).

To investigate the role of FGGY in metabolic-immune regulation, we assessed the *FGGY* isoform pattern in relation to a panel of T cell-specific genes (Figure 1B). Among these genes, *ENTPD1* (CD39), a key ectonucleotidase that suppresses T cell activation by converting immunogenic ATP into immunosuppressive adenosine, emerged with *FGGY* isoform-specific association. The alternatively spliced *FGGY*-204 displayed a positive correlation with *ENTPD1* expression, whereas the canonical *FGGY*-201 was negatively correlated. This antagonistic relationship suggests that *FGGY* isoforms can act as functional mediators, taking part in shaping the immunogenic landscape of HCC tumors (Figure S1D). Moreover, *FGGY*-204 is negatively correlated with *CD8A* expression, a gene encoding the alpha chain in the core CD8 glycoprotein, which is essential for cytotoxic T cell function, suggesting a further coupling of *FGGY-*204 expression and the observed depletion of cytotoxic T cells in the tumor (Figure S1E). Additionally, the *FGGY*-204 expression level in MASLD livers is negatively correlated with key liver injury markers (alanine aminotransferase [ALT], aspartate aminotransferase [AST], and the ALT/AST ratio). A relationship with liver function is not observed for the canonical *FGGY-*201 transcript (Figures 1B and S1G), suggesting that *FGGY*-204 is specifically associated with hepatocyte integrity and/or takes part in protection against metabolic rearrangements in MASLD, thereby showing a distinct role of *FGGY*-204 from the canonical isoform.

The alternatively spliced isoform *FGGY*-204 contains an extra exon 12 (hg38: 59641280-59641351; 407-MRTTGYLYIPALAALHSPSSLLSPQ-431), encoding a 25-amino acid sequence that is absent from other FGGY isoforms, including the canonical FGGY-201. This peptide is distal to the active site of FGGY, suggesting minimal impact on the core protein catalytic function (Figure 1D). The exclusive FGGY-204 exon 12 was validated by multiple high-confidence tandem MS (MS/MS) spectra, which identified a unique 27-amino acid peptide (TTGY-*LYIPALAAL-*HSPSSLLSPQVTGLK) derived from exon 12, as well as the immunogenetic neoepitope LYIPALAAL that binds two HLA alleles (Table S1, core epitope identification). This epitope demonstrated efficient proteasomal cleavage, adequate TAP transport, positive immunogenicity, and favorable overall HLA presentation (Figures 1E, 1F, S1G, and S1H). Structural modeling revealed a potential T cell recognition (Figure 1G), in which an arginine residue from the TCR α-chain interacts with the N-terminal region of the neopeptide, while the β-chain leucine engages the second leucine of the neopeptide through direct backbone hydrogen bonding and hydrophobic side-chain stacking. Additionally, four cryptic peptides associated with exon 12 were also detected and validated in multiple proteomics datasets spanning diverse cancer types, demonstrating a wider tumor-specific protein expression (Table S1, proteomic validation). Moreover, FGGY exon 12 is insignificantly included in normal livers, further supporting the tumor specificity of its derived peptides (Figure S1I).

This core epitope represents an HLA-presented target, not found in the Immune Epitope Database (IEDB), HLA binding databases (hla_2020.12), and the Peptides for Cancer Immunotherapy Database (PCI-DB). However, while a shorter nested peptide (IPALAA) derived from Importin-4 is detectable in healthy tissues via PCI-DB analysis, the complete exon 12-derived epitope of FGGY-204 is exclusively tumor specific. Supporting this high degree of tumor specificity, *in silico* assessment confirmed no significant sequence similarity to other human peptides presented by common MHC alleles, minimizing potential cross-reactivity concerns (Table S1, IEDB analysis). Finally, exon 12 is a primate-specific sequence with no conserved murine orthologs, presenting a challenge for *in vivo* modeling while underscoring its potential as a human-specific therapeutic target. Together, these observations prompted us to further investigate *FGGY* isoforms and the regulation that may enable tumor-specific inclusion of exon 12 in FGGY-204.

### *FGGY* chromatin state dynamics is tissue-specific and regulated by tumor cell epigenetic repression

**Figure 2:**
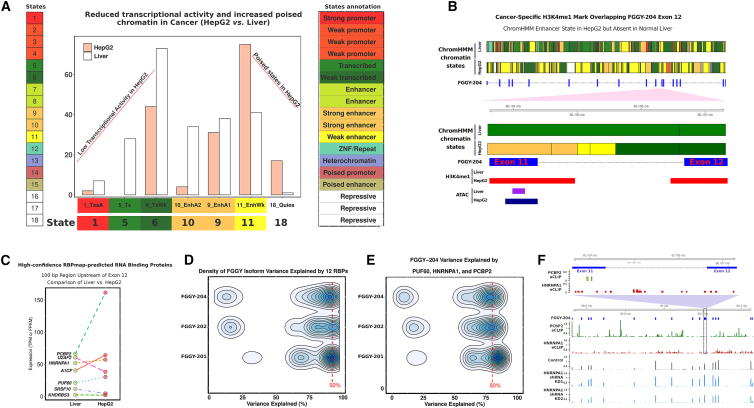
Chromatin and post-transcriptional regulation of FGGY isoforms reveals tissue-specific epigenetic silencing and RBP-mediated control of FGGY-204 in HCC (A) Comparison of selected chromatin states at the *FGGY* locus between liver and HepG2. Bar plot shows the frequency of key ChromHMM states overlapping the *FGGY* gene region (TSS +2 kb) in normal liver (E066) and HepG2 liver tumor cells (E118). Active states are enriched in liver, whereas HepG2 exhibits higher proportions of weak or repressive states. (B) Genomic tracks showing chromatin states, *FGGY*-204 exons 11 and 12, H3K4me1 and H3K27ac, and ATAC-seq accessibility in liver and HepG2 cells across the specified *FGGY* locus (±300 bp). (C) Expression levels of high-confidence RBPmap-predicted RNA-binding proteins in liver and HepG2 cells. Line plot shows gene expression levels across tissues for RBPs predicted to bind within 100 bp upstream of exon 12 of FGGY-204. (D) Variance explained in expression of FGGY isoforms by 12 filtered RNA-binding proteins (RBPs). (E) RBPs show strong binding for *FGGY*-204, suggesting isoform-specific regulation. (F) Genome browser-style visualization showing eCLIP enrichment for RNA-binding proteins PCBP2 (dark green) and HNRNPA1 (light red) across the *FGGY* transcript variant ENST00000371218 (*FGGY*-204). RNA sequencing (RNA-seq) signal from control shRNA treatment (non-target-BGHLV20) and HNRNPA1 knockdown (HNRNPA1-BGHLV20) in HepG2 cells is also shown. The top panel displays signal across the full *FGGY*-204 transcript, while the bottom panel zooms in on exons 11 and 12, along with intron 11, to highlight localized RBP binding. Peaks were derived from narrowPeak files, and signal intensity is represented as histogram bars along genomic coordinates (hg19). Exonic regions are shaded in sky blue.

To map tissue-specific chromatin dynamics at the *FGGY* locus across diverse human tissues and cell lines, we applied the 18-state multivariate hidden Markov model (ChromHMM-18). In liver, the *FGGY* locus displays strong transcriptional and promoter-associated chromatin states (Figures 2A and S2A), whereas HepG2 (liver tumor model) shows a shift toward a weaker or poised promoter state, consistent with epigenetic repression in cancer. Active enhancers (state 10 EnhA2) are notably silenced in HepG2, while weak enhancers (state 11 EnhWk) are increased, reflecting inactive regulatory primed elements. This shift reflects loss of enhancer fidelity and increased condensation of heterochromatin, presenting a quiescent cancer-associated chromatin remodeling at the *FGGY* locus.

Despite ongoing epigenetic remodeling in HepG2, there are marked changes in the expression of histone 3 lysine 27 acetylation (H3K27ac)-associated enzymes with reduced levels of HDAC1/2, EP300, and BRD2/3, alongside increased expression of BRD4 and CREBBP (Figure S2B). This epigenetic imbalance disrupts an overall histone acetylation homeostasis contributing to an altered chromatin landscape and gene regulatory environment characteristic of cancer cells. Notably, H3K27ac, in conjunction with H3K4me1 and H3K9ac, remains enriched at active enhancer and promoter states (state 9 EnhA1; state 11 EnhWk, state 6 TxWk), highlighting its pivotal role in transcriptional regulation at the *FGGY* locus (Figures S2C and S2D).

In normal liver, hepatocyte nuclear factor 4a (HNF4A) and yin yang 1 (YY1) co-localize with H3K27ac and open chromatin regions (shown by ATAC-seq), reinforcing canonical liver-specific *FGGY* transcription (Figure S2E). In contrast, the HepG2 chromatin landscape retains forkhead box protein A1 (FOXA1) binding but loses HNF4A and YY1 occupancy, accompanied by diminished H3K27ac enrichment. Notably, YY1 binding is absent beyond the promoter-proximal region, demonstrating a broader regulatory role in transcription. In liver tissues, 16 ATAC peaks colocalize with YY1, whereas in HepG2 only 5 peaks remain, consistent with a substantial loss of YY1 occupancy. This regulation is observed across both the canonical *FGGY*-201 transcript and the tumor-specific *FGGY*-204 isoform, indicating that diminished YY1 binding contributes to the reduced canonical *FGGY* expression in HepG2 tumor cells.

### Integrated epigenetic and transcriptional regulation of *FGGY* isoforms

To further define the basis for tumor-specific *FGGY*-204 expression, we characterized the regulatory landscape of exon 12 in HepG2 tumor cells using publicly available epigenomic datasets from the ENCODE and Roadmap Epigenomics consortia. The exon 12 region is devoid of active chromatin marks, which is shown by the absence of chromatin accessibility (DNase-/ATAC-seq, ENCODE data) and H3K27ac chromatin immunoprecipitation sequencing (ChIP-seq) peaks, as well as lack of enhancer activity based on CAGE-seq data (FANTOM5). These features suggest a poised latent genomic state in HepG2. This is further supported by specific H3K4me1 enrichment, and a corresponding weak enhancer state (state 11 EnhWk) shown in the Roadmap ChromHMM-18 model, which is contrasted by the weak transcriptional state (state 6 TxWk) observed in normal livers (Roadmap Epigenomics; Figure 2B). Splicing enhancer analysis further identified strong exonic splicing enhancer motifs within exon 12, supporting its latent regulation (Figure S2F).

This poised chromatin context establishes a permissive state for RNA-binding protein (RBP)-mediated control of exon 12 inclusion. As such, we identified a tumor-associated shift in the RBP regulatory network, which is inversely correlated between HepG2 and normal livers, and drives *FGGY*-204 expression variance (Figures 2C–2E; Table S2). To further quantify the contribution of individual RBPs to isoform regulation, variance decomposition analysis demonstrated that specific RBPs account for a significant proportion of FGGY isoform expression variance (Figure S3A). High-resolution mapping of RBP-binding sites by eCLIP in HepG2 confirmed binding of HNRNPA1 specifically enriched in *FGGY* intron 11 (Figure 2F). Functional validation demonstrated that small hairpin RNA (shRNA)-mediated knockdown of *HNRNPA1* increased *FGGY*-204 expression (Figure S3B), pinpointing it as an intronic splicing silencer for exon 12. Together, these data reveal that tumor-specific *FGGY*-204 activation is driven by a coordinated chromatin-RBP mechanism, where release of HNRNPA1-mediated repression unlocks exon 12 inclusion in the malignant state.

**Figure 3:**
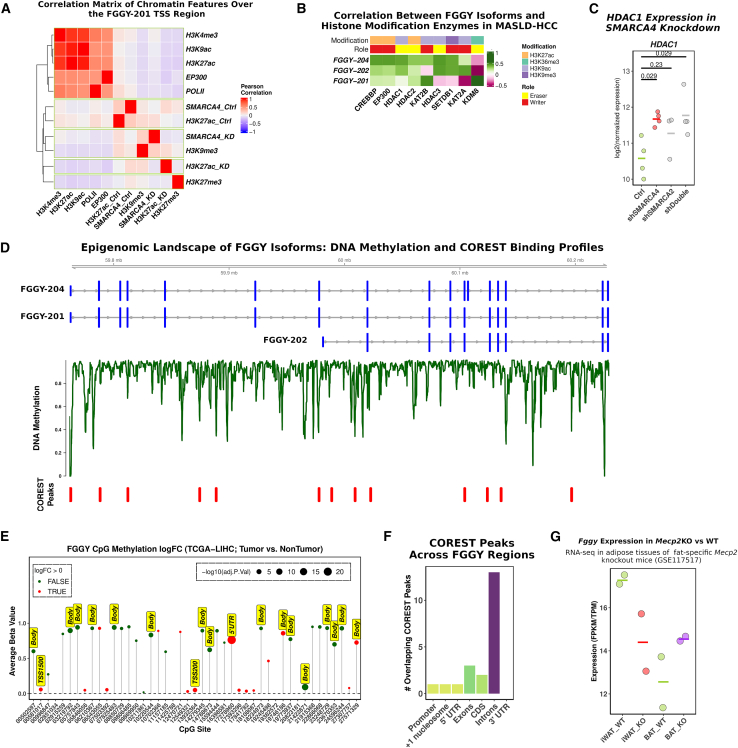
Epigenetic and transcriptional regulation of *FGGY* isoforms (A) Isoform-specific chromatin landscapes at the *FGGY*-201 locus. Heatmaps of Pearson correlations among ChIP-seq signals for active (H3K9ac, H3K27ac, H3K4me3, POLII, and EP300) and repressive (H3K27me3 and H3K9me3) marks at the ±5 kb region surrounding the transcription start site (TSS) of *FGGY*-201. *FGGY*-201 promoters show strong correlations among active marks, indicative of transcriptionally engaged chromatin, which is partially disrupted upon SMARCA4 knockdown. Repressive marks show weak or negative correlations with active marks, reflecting mutually exclusive chromatin states. (B) Correlation heatmap of epigenetic histone modifications at *FGGY* regulatory regions. Spearman correlations between active (H3K27ac, H3K9ac, and H3K36me3) and repressive (H3K9me3) marks were calculated in our MASLD-HCC cohort. Warm colors represent positive correlations, while cool colors represent negative correlations, highlighting coordinated or antagonistic chromatin regulation at *FGGY* loci. (C) Expression of HDAC1 in HepG2 cells upon SMARCA4 knockdown (GSE102560). Jitter plots show individual log2-normalized expression values under control (Ctrl), SMARCA4 knockdown (shSMARCA4), SMARCA2 knockdown (shSMARCA2), and double knockdown conditions. Red bars indicate mean expression per condition. Statistical significance was assessed using pairwise comparisons with Wilcoxon tests. HDAC1 expression is significantly upregulated in SMARCA4 knockdown, consistent with enhanced histone deacetylation at target promoters. (D) Integrated visualization of the FGGY locus showing smoothed DNA methylation levels, gene structure, COREST binding peaks, and highlighted regions of interest. The plot illustrates the relationship between DNA methylation and COREST occupancy across the gene body and regulatory elements. (E) Differential CpG methylation at the FGGY locus in TCGA-LIHC tumors versus normal tissue. Bars represent the log fold change (logFC) of significantly altered CpG sites (adj. *p* < 0.05), with color indicating direction and magnitude of change (yellow/green: hypomethylated; dark red: hypermethylated). CpG sites are annotated by genomic context and regulatory feature, highlighting differential methylation patterns across promoters, gene bodies, and other regulatory regions. (F) Number of CoREST ChIP-seq peaks overlapping *FGGY* promoter, exons, and introns. (G) Expression of *FGGY* isoforms in MECP2 CRISPR knockdown versus wild-type Huh7 HCC cells. Jittered points show individual sample expression values across conditions (GSE176935) with each facet representing a distinct *FGGY* isoform, highlighting isoform-specific transcriptional changes upon MECP2 perturbation.

Having established isoform-specific regulation at the *FGGY* locus, we next characterized its underlying epigenomic landscape by integrative analysis of histone marks using ChIP-seq data, highlighting isoform-specific chromatin states and a central role for SMARCA4 in maintaining these states (Figures S3C and S3D). The canonical *FGGY*-201 promoter resides in a transcriptionally active chromatin state, evidenced by strong positive correlation between active histone marks (H3K9ac, H3K27ac, H3K4me3) and POLII occupancy, while repressive marks showed a negative correlation (Figure 3A). This active state is highly dependent on SMARCA4, as SMARCA4 knockdown significantly disrupts the chromatin dynamics by increasing HDAC1 expression and results in loss of active marks, indicating a shift toward a repressive chromatin state (Figures 3A–3C). The *FGGY*-204 promoter also showed features of active chromatin but with an intermediate reliance on SMARCA4 (Figure S3D). In contrast, the *FGGY-202* promoter exhibited a repressed chromatin landscape that is unchanged by SMARCA4 depletion (Figure S3C). These chromatin states are dynamically shaped by the antagonistic activity of histone-modifying enzymes (KAT2A, HDAC1, SUV39H1, and SETDB1), which are dysregulated in MASLD-HCC (Figure 3B). SMARCA4 regulation is critical for balancing this isoform-specific epigenetic equilibrium toward activation at the *FGGY-*201 promoter. SMARCA4 loss tips the balance, promoting a repressive chromatin state that contributes to the transcriptional silencing of the canonical *FGGY-*201 isoform observed in MASLD-HCC tumors.

### CoREST-mediated repression fine-tunes *FGGY* chromatin states

To further understand the regulation at the *FGGY* locus, we assessed DNA methylation patterns and repressor complex occupancy. Whole-genome bisulfite sequencing data derived from liver samples were used to assess DNA methylation patterns across the *FGGY* locus (Roadmap Epigenomics). These data revealed consistently high levels of DNA methylation across the gene body, with loss of methylation at the promoter (Figure 3D), suggesting active transcription of *FGGY*. Moreover, reference data from HepG2 histone profiling showed a poised/bivalent chromatin configuration at the *FGGY* promoter (Figures 2A and S2A). Also. tumor tissues exhibited markedly elevated DNA methylation levels compared with non-tumoral tissue, particularly within exonic and intronic regions (Figure 3E).

To assess potential transcriptional repressors, we analyzed ChIP-seq data from 208 transcription factors in HepG2 cells,^21^ predominantly displaying limited binding peaks within the *FGGY* locus. However, the corepressor of repressor element 1 silencing transcription factor (CoREST) complex showed a strong binding with 13 distinct peaks distributed across the *FGGY* gene body (three located within exons, one peak at the promoter, and 9 in introns; Figures 3D and 3F). CoREST is a component of the BHC (BRAF-HDAC) complex, which facilitates transcriptional silencing through recruitment of histone demethylases and methyl-CpG-binding proteins (MeCP2). As such, co-localization of CoREST peaks with hypermethylated regions suggests a concerted silencing that fine-tunes *FGGY* isoform usage. MeCP2 recruits the CoREST complex to methylated DNA regions, and thus, MeCP2 knockout^22^ was shown to either lead to decreased *FGGY* expression in white or increased expression in brown adipose tissues (Figure 3G), suggesting that the MeCP2-CoREST complex exerts a tissue-specific regulatory role on FGGY corresponding to the liver.

### *SMARCA4*-*KDM8*-*CDK9* axis enforces isoform-specific transcriptional control

**Figure 4:**
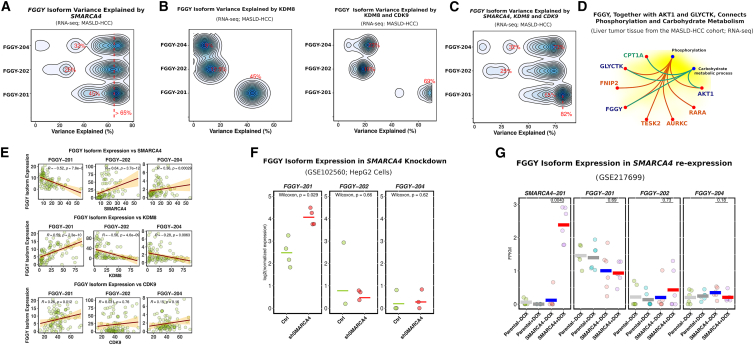
Multimodal analysis reveals isoform-specific regulation of FGGY by SMARCA4, KDM8, and CDK9 within a broader metabolic and transcriptional context (A) Partial least squares (PLS) linear regression analysis shows that SMARCA4 accounts for up to 65% of the variance in expression of *FGGY*-201, *FGGY*-202, and *FGGY*-204. (B) Isoform-specific regulation of *FGGY* by KDM8 and CDK9. Density distribution plots show the proportion of variance in expression of *FGGY*-201, *FGGY*-202, and *FGGY*-204 explained by KDM8. *FGGY*-201 exhibits a stronger association with KDM8 compared to the other isoforms. (C) Combined variance in *FGGY* expression explained by SMARCA4, KDM8, and CDK9. (D) Network representation of co-expression pathways involving FGGY and associated genes. Nodes represent genes, colored by their annotated pathway membership. Edges denote shared pathway involvement, with edge colors indicating specific biological processes. The circular layout highlights distinct non-tumoral interactome patterns and context-specific clustering. (E) *FGGY* isoform expression in relation to SMARCA4, KDM8, and CDK9. Scatterplots showing the expression levels of *FGGY* isoforms plotted against *SMARCA4, KDM8*, and *CDK9* expression. (F) *FGGY* isoform expression upon SMARCA4 knockdown. (G) *FGGY* isoform expression upon SMARCA4 re-expression. Expression levels of *FGGY* isoforms across four conditions with doxycycline (DOX) control.

The canonical *FGGY*-201 isoform is repressed by a coordinated mechanism involving *SMARCA4*, *KDM8* (*JMJD5*), and *CDK9*, as defined by transcription start site and transcriptional regulator analyses (Figures 4A–4D and S4A–S4G). SMARCA4 emerges as a master regulator of FGGY isoform balance, coordinating repression of the canonical *FGGY-*201 transcript, which was confirmed by *SMARCA4* knockdown and re-expression experiments (Figures 4F, 4G, and S5A–S5C). This repression is functionally linked to KDM8, a gene SMARCA4 regulates (Figures S5B and S5C).

KDM8 and CDK9 synergistically cooperate, controlling transcriptional pausing and elongation.^23^ Together, these factors account for nearly 70% of the variance observed in *FGGY-*201 expression, which is further supported by PLS modeling showing *KDM8* alone distinguishes the canonical from alternative isoforms (Figures 4B and 4C). As such, FGGY is located within a broader co-expression network enriched for phosphorylation and metabolic processes consistent with KDM8/CDK9 transcriptional control. This defines a hierarchical regulatory cascade where SMARCA4 establishes the chromatin context, KDM8 modulates histone methylation, and CDK9 executes Pol II RNA dynamics and transcriptional control repressing *FGGY-*201.

### Spatial isoform-specific and metabolic regulation of *FGGY* in HCC

FGGY expression is enriched in hepatocytes, with distinct isoform- and temporospatial-specific regulation in the normal liver and HCC tumors (Figures S6A–S6D). To understand the functional consequences of this regulation, we performed untargeted metabolomics to assess carbohydrate and pentose phosphate pathway (PPP) intermediates. In healthy liver, spatial transcriptomics revealed that *FGGY* is restricted to periportal (zone 1) hepatocytes responsible for oxidative metabolism. In HCC, this zonation is lost, and *FGGY* expression becomes broadly elevated across malignant hepatocytes (Figure S6D). Metabolically, this rewiring is reflected in a significant decrease in key PPP intermediates, including the putative FGGY products ribulose-5-phosphate and xylulose-5-phosphate (Figures S6E–S6F). Isoform-specific analysis showed downregulation of the full-length, catalytic *FGGY*-201 isoform in tumors, while total *FGGY* mRNA increased due to the truncated, non-catalytic *FGGY*-202 isoform (Figure S1B). Correlation analysis linked *FGGY*-201 expression specifically to several carbohydrate metabolites, including α-D-glucose and D-ribose 5-phosphate, suggesting that FGGY-201 coordinates glycolytic and PPP fluxes (Figures S6G–S6I). Finally, NetPhos analysis of FGGY-204 neoantigen isoform revealed multiple predicted phosphorylation sites within its unique exon 12 sequence, suggesting that it may undergo phosphorylation-dependent modulation of its stability or signaling interactions, thereby adding an additional layer of post-translational control (Figure S6C).

### *FGGY*-204 expression highlights immune cell trafficking bottleneck across treatment time points in HCC

**Figure 5:**
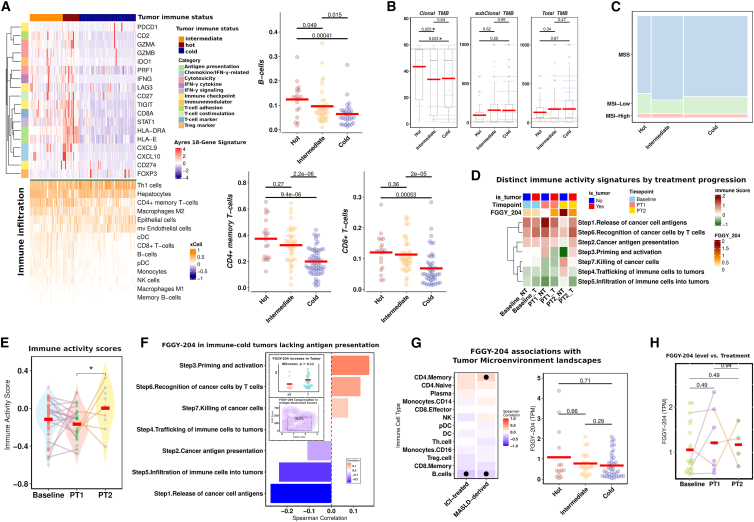
Immune landscape, tumor mutational burden, microsatellite instability status, and *FGGY*-204 expression across clusters and treatment cycles (A) Immune-related gene signatures and cell fractions across clusters. Heatmap of the 18-gene immune checkpoint signature (top), annotated by functional categories, and corresponding xCell-derived immune cell fractions (bottom). Patients are shown in columns and genes in rows; expression values are standardized (centered and scaled within each column). Rows and columns were clustered using Ward’s method. Immune cell infiltration was estimated using xCell, with cluster classifications shown in annotation tracks. Beeswarm and boxplots showing relative abundances of CD4^+^ memory T cells, CD8^+^ T cells, and B cells across hot, intermediate, and cold clusters. Red bars indicate group means. Pairwise differences were evaluated with Wilcoxon tests; significant *p* values are annotated. (B) Tumor mutational burden (TMB) across clusters. Dot and boxplots showing TMB fractions in hot, intermediate, and cold clusters. Red bars indicate group means. Pairwise comparisons were assessed using Wilcoxon tests, with *p* values indicated; significant differences are marked with ∗. (C) Distribution of microsatellite instability/stable (MSI/MSS) status across assigned clusters. Mosaic plot illustrating the proportional distribution of MSI categories (MSI-high, MSI-low, MSS) within each cluster. Box widths correspond to cluster sizes, and colored segments indicate relative MSI proportions. (D) Heatmap of average ssGSEA-derived immune scores showing distinct immune activity signatures across tumor status and treatment progression. Rows represent immunophenotyping, and columns represent averaged sample groups stratified by tumor status and treatment stage. Color scale ranges from low (dark green) to high (dark red) immune activity. (E) Immune activity scores at baseline and treatment cycles PT1 and PT2. Violin plots show the distribution of scores at each time point, with red bars indicating group means. Points represent individual patients, and paired segments connect matched samples across time points. *p* values from Wilcoxon tests are shown for significant pairwise comparisons. (F) Bar plot showing Spearman correlations between *FGGY*-204 expression and steps of the immunophenotyping cycle, with steps ordered by correlation magnitude; positive (red) and negative (blue) correlations indicate direct and inverse associations, respectively. Insets in the top left corner include a beeswarm plot demonstrating higher *FGGY*-204 expression in tumor versus matched non-tumor tissue, with individual values, group means (red bars), and *p* value indicated, and a density contour plot illustrating the relationship between *FGGY*-204 expression and MHC score in tumor samples, where contours highlight areas of higher sample density. (G) Heatmap of Spearman correlations between *FGGY*-204 isoform expression and immune cell type abundances in ICI-treated versus MASLD-derived HCC without treatment (left). Colors indicate the strength and direction of correlations (blue, negative; red, positive; white, none), with numeric correlation values displayed on each tile for precision. *FGGY*-204 expression across clusters (right). Beeswarm plots showing *FGGY*-204 transcript levels (TPM) across clusters. Red bars indicate group means. Pairwise differences were assessed using Wilcoxon tests; significant *p* values are annotated. (H) *FGGY*-204 expression increases across treatment time points in approximately half of individuals. Beeswarm plots show individual patient values at PT1 and PT2. Red bars denote group means, and paired segments connect matched samples over time. *p* values from pairwise comparisons are indicated.

To investigate the relationship between FGGY-204 and the tumor immune microenvironment (TIME), we first characterized the immune landscape in an immunotherapy-treated HCC cohort (NCI CLARITY study).^24^ Unsupervised clustering of tumors based on an 18-gene IFN-γ signature revealed three distinct immune groups: “hot,” “intermediate,” and “cold” (Figure 5A). Hot tumors were further characterized by elevated expression of chemokines (CXCL9 and CXCL10) and HLA class II genes and increased infiltration of CD4^+^ and CD8^+^ T cells as well as B cells (Figures 5A and 5B). The immunologically active tumors were also enriched for clonal tumor mutational burden (Figure 5C). Immune phenotyping using single sample gene set enrichment analysis (ssGSEA) further confirmed distinct immune activity signatures across tumor status and treatment progression, with heatmap visualization revealing differential immune activation between tumor stages and therapy time points (Figure S7A).

Longitudinal analysis using the TIP algorithm showed that overall immune activity increased significantly over the course of therapy, peaking at the second on-treatment time point (PT2) (Figures 5D and 5E). Having established this dynamic immune context, we then examined the role of *FGGY*-204. Intriguingly, *FGGY*-204 expression was negatively correlated with step 1 (antigen presentation) of the cancer-immunity cycle (r = −0.27; Figures 5F and S7B), suggesting it may be preferentially expressed in tumors with inherent defects in antigen release or presentation.

Next, we assessed the relationship between *FGGY*-204 and specific immune cell subpopulations. This revealed a complex and context-dependent relationship. In the treatment-naive MASLD-HCC cohort, total *FGGY* expression showed positive correlation with CD4^+^ memory T cells, whereas in the ICI-treated NCI-CLARITY cohort, *FGGY-*204 is significantly negative correlated with B cells (Figure 5G). Moreover, *FGGY-*204 levels were found to be highest in immunologically “hot” tumors (Figure 5H). As such, mixed-effects modeling identified *FGGY-*204 as a dynamically regulated transcript over the course of immunotherapy, grouping the isoform with established HCC marker alpha-fetoprotein and karyopherin subunit alpha 2 (Figure S7C). Additionally, we identified four somatic missense mutations in *FGGY* (p.G105W, p.V191M, p.A290V, and p.G330D) specifically in ICI-treated patients, with two mutations (p.G105W and p.V191M) representing clonal events. Integrating somatic variant data with immune cluster assignments revealed that the four mutations are enriched in the context of immune-cold tumors, suggesting a possible involvement for mutated FGGY in reduced immune infiltration or evasion.

Together, these findings emphasize FGGY-204 as a context-dependent immune modulator, associated with CD4^+^ T cell presence and dynamic shifts in the TIME during immunotherapy.

## Discussion

Alternative splicing has emerged as an important source of tumor-specific neoantigens.^25^^,^^26^ Previous studies have demonstrated that splicing-derived peptides can be presented by MHC molecules and elicit T cell responses, highlighting their therapeutic potential in cancer immunotherapy.^27^ These findings have motivated the development of neoantigen-based vaccine strategies targeting both mutation-derived and splicing-derived epitopes.^28^^,^^29^

Our study identifies FGGY-204 as human-specific neoantigen derived from alternative splicing in HCC. Its repression is ensured by a multi-faceted mechanism, involving a poised chromatin state and active silencing by the splicing factor *HNRNPA1*. The de-repression of this regulatory axis in HCC exposes a peptide sequence that contributes to the immune-evasive nature of these tumors.

This study identifies two potential translational avenues. The exon 12 peptide represents a candidate splicing-derived neoantigen that could be explored in vaccine strategies. Additionally, modulation of splicing regulators such as HNRNPA1 may represent a hypothetical approach to enhance antigen presentation, but its feasibility and safety require extensive preclinical investigation.

We reveal a dichotomy in FGGY regulation: the canonical metabolic isoform (*FGGY*-201) is controlled transcriptionally by a *SMARCA4*-*KDM8*-*CDK9* axis, while the neoantigen-encoding isoform (*FGGY*-204) is controlled post-transcriptionally by chromatin context and *HNRNPA1*-mediated splicing. This contrast allows for an FGGY-related tumor-specific regulation.

The dynamic relationship between FGGY-204 expression and the TIME, particularly its shift across ICI treatment cycles, indicates that the exon 12-derived neoantigen functions in a context-dependent manner rather than as a constitutively immunogenic target. Its activity likely reflects treatment- and ecosystem-specific immune dynamics, including fluctuations in T cell priming and checkpoint-mediated suppression. Accordingly, FGGY-204 may serve as a therapeutic opportunity, particularly within rational combination strategies aimed at reshaping immune-cold tumors.

MS data demonstrate that the exon 12-derived peptide is presented in the context of HLA class I molecules, establishing its potential for CD8^+^ T cell recognition at the level of antigen presentation. However, immune correlation analyses reflect ecosystem-level immune composition rather than antigen-specific T cell responses. The observed association with CD4^+^ T cells, therefore, underscores broader immune dynamics within the tumor microenvironment and does not exclude the possibility of CD8-mediated recognition. Indeed, lack of correlation with bulk CD8^+^ T cell abundance likely reflects the complexity and context-dependent nature of immune engagement in HCC, including the presence of low-frequency antigen-specific clones and checkpoint-mediated T cell dysfunction.

### Limitations of the study

Several limitations should be acknowledged. The initial identification of the alternatively spliced *FGGY*-204 relied on a prediction of peptide-MHC binding. The direct proof of immunogenicity through the isolation of FGGY-204-specific T cell clones from patients is currently missing. This limitation was bypassed by demonstrating *FGGY*-204 correlation with an immune-suppressed microenvironment and strong MS/MS validation confirming the presence of the FGGY-204 neopeptide at the protein level in human tumors. Finally, the therapeutic potential of targeting *HNRNPA1* requires a more comprehensive assessment of potential off-target effects, as systemic splicing modulation could inadvertently expose cryptic neoantigens in healthy tissues. Addressing these points in future studies will be critical for translating these findings into clinical applications.

While our findings highlight a promising mechanism and therapeutic opportunities, further functional *in vivo* validation, assessment across diverse HCC cohorts, and exploration of potential off-target effects of splicing modulation will be essential to confirm the safety and efficacy of these approaches. In conclusion, we have unraveled a complex mechanism that controls and safeguards a potential neoantigen. The failure of this repressive mechanism in tumor cells represents a therapeutic actionable vulnerability in HCC.

## Resource availability

### Lead contact

Requests for further information and resources should be directed to and will be fulfilled by the lead contact, Jesper B. Andersen (jessper.andersen@bric.ku.dk).

### Materials availability

This study did not involve the generation of any materials.

### Data and code availability

• RNA sequencing data of MASLD^30^ have been deposited in the Sequence Read Archive (SRA) under accession number PRJNA1185558 and in the Gene Expression Omnibus (GEO) under accession number GSE281797 and are publicly available as of the date of publication. Accession numbers are listed in the key resources table.
• MASLD clinical data, processed omics datasets, and analysis code are available at https://github.com/SLINGhub/MASLD_dual_omics and archived at SLINGhub (2025).
• The RNA-seq and metabolic data of MASLD-HCC.^18^ Data used in the analysis is represented in Table S3.
• This paper does not report original code. Any additional information required to reanalyze the data reported in this paper is available from the lead contact upon request.

## Acknowledgments

The authors would like to thank the patients and their families for allowing research access to their samples. Also, we are grateful for access to all public datasets. The results are in part supported by data generated by the TCGA Research Network (https://www.cancer.gov/tcga). The laboratory of J.B.A. is supported by competitive funding from the 10.13039/501100009708Novo Nordisk Foundation (0058419, 220C0074956, and 0085704), 10.13039/100008363Danish Cancer Society (R167-A10784, R278-A16638, and R368-A21455), and Independent Research Fund Denmark for Medical Research (4183-00118A and 1030-00070B). L.N.Z would like to express her gratitude to Qing Zhao (Lexie) and Profs. Mikael Bjorklund, Hyungwon Choi, Matthew J. Watt, Philipp Kaldis, and Patrik Midlöv. Some figures were created using BioRender.com.

## Author contributions

Conceptualization, L.N.Z and J.B.A; methodology, L.N.Z. and J.B.A.; investigation, L.N.Z. and J.B.A.; writing – review and editing, L.N.Z. and J.B.A; funding acquisition, J.B.A.

## Declaration of interests

J.B.A declares consultancies for Flagship Pioneering, QED Therapeutics, and AstraZeneca. J.B.A has received funding from the Incyte Corporation and ADCendo but is not related to this study.

## Declaration of generative AI and AI-assisted technologies in the writing process

ChatGPT or other LLMs were not used to assist the authors in this manuscript.

## STAR★Methods

### Key resources table

**Table:** 

REAGENT or RESOURCE	SOURCE	IDENTIFIER
**Deposited data**		

MASLD cohort	Sequence Read Archive (SRA)	PRJNA1185558
MASLD cohort	Gene Expression Omnibus (GEO)	GSE281797
Immune Epitope Database (IEDB)	IEDB	https://www.iedb.org/
HLA binding database	NetMHCpan (hla_2020.12)	https://hla-ligand-atlas.org
Peptides for Cancer Immunotherapy Database (PCI-DB)	PCI-DB	https://pci-db.org/
FANTOM5 CAGE dataset	FANTOM Consortium	https://fantom.gsc.riken.jp/
GeneCards	Weizmann Institute	https://www.genecards.org/

**Software and algorithms**		

IsoformSwitchAnalyzeR	Bioconductor	https://bioconductor.org/packages/release/bioc/vignettes/IsoformSwitchAnalyzeR/inst/doc/IsoformSwitchAnalyzeR.html
DEXSeq	Bioconductor	https://bioconductor.org/packages/release/bioc/vignettes/DEXSeq/inst/doc/DEXSeq.html
NetMHCpan	Technical University of Denmark	https://services.healthtech.dtu.dk/
RBPmap	Weizmann Institute	http://rbpmap.technion.ac.il/
ESEfinder	Cold Spring Harbor Laboratory	https://krainer01.cshl.edu/cgi-bin/tools/ESE3/esefinder.cgi
ChEA3	Ma’ayan Lab	https://maayanlab.cloud/chea3/
TIP (Tumor Immune Profiling)	TIP webserver	https://doi.org/10.1158/0008-5472.CAN-18-0689
xCell	GitHub	https://github.com/dviraran/xCell
CIBERSORTx	Stanford University	https://cibersortx.stanford.edu/
WGCNA	R package	https://cran.r-project.org/web/packages/WGCNA/WGCNA.pdf
BWA-MEM	GitHub	https://bio-bwa.sourceforge.net/
Funcotator	Broad Institute	https://gatk.broadinstitute.org/hc/en-us/articles/360037224432-Funcotator
Picard	Broad Institute	https://broadinstitute.github.io/picard/
GATK (HaplotypeCaller, Mutect2)	Broad Institute	https://gatk.broadinstitute.org/hc/en-us
MANTIS	GitHub	https://github.com/PedroMTQ/mantis

### Methods

#### Study participant details

Our perspective patient samples are from two cohorts: a MASLD cohort (42 healthy controls, 66 individuals with MASLD, and 11 with MASH)^31^ and a MASLD-HCC cohort (120 paired tumor and adjacent non-tumor tissue samples, representing disease progression from MASLD to HCC).^32^ The MASLD study protocol conforms to the ethical guidelines of the 1975 Declaration of Helsinki and was approved by Etikprövningsmyndigheten (Dnr 2024-07917-01) in Sweden. MASLD-HCC was performed following individual patient consent, local institutional review board approvals (IORG0003254; IRB00003888), and assessed by the Committee on Health and Research Ethics for the Capital Region of Denmark for use of archival material (no. H-4-2016-FSP, 17029679). All patient datasets were anonymized. This study was not designed to evaluate sex-specific differences, and sex was not included as a variable in downstream analyses. Therefore, potential sex-associated effects cannot be excluded.

#### Public datasets

External and public datasets, include gene expression and chromatin accessibility [GSE73491,^33^ GSE153322,^23^ GSE275694^34^]; transcription factor and ChIP-seq [GSE104247, GSM935579, GSM935511^22^; GSE198517,^35^ GSE153576, GSE153576^36^; GSM733638, GSM1220298, GSM733743, GSM733685, GSM945211, GSM1003519, GSM733641, GSM733694, GSM798321, GSM733693, GSM733737, GSM945182, GSM537697, GSM537698, GSM733754, GSM945231, GSM733774]; Proteomics PRIDE database (PXD006512)^37^ and data from the National Translational Science Network of Precision-Based Immunotherapy for Primary Liver Cancer (phs003074.v1.p1)].^24^ All datasets were integrated and analyzed using standardized pipelines, with metadata harmonized across studies for downstream analyses including transcriptional regulation, chromatin accessibility, histone modification profiling, and proteomic validation.

#### Isoform switch analysis

Isoform switching was analyzed using *IsoformSwitchAnalyzeR*,^38^^,^^39^ with differential isoform usage quantified via DEXSeq.^40^^,^^41^ Predicted functional consequences, such as alterations in coding potential, protein domains, and subcellular localization, were assessed to evaluate the impact of switching events. Significant isoform switches were defined by an absolute ΔIF ≥0.1 and a false discovery rate (FDR) ≤ 0.05.

#### 10-Fold cross-validation partial least squares (PLS) regression

PLS regression was used to evaluate the relationship between transcription regulator expression and variability in FGGY isoform expression. The dataset was partitioned into 10 folds for cross-validation, and the variance explained by each transcription regulator was quantified using R-squared values.

#### Mass spectrometry analysis of FGGY peptides

Mass spectrometry was performed to validate tumor-specific FGGY peptides derived from exon 12. The core epitope (residues 412-420; YLYIPALAA) has a monoisotopic mass of 1075.54 Da, an average mass of 1076.65 Da, and a theoretical isoelectric point (pI) of 5.52. The full-length exon 12 peptide (residues 407-431; MRTTGYLYIPALAALHSPSSLLSPQ) has a monoisotopic mass of 2686.42 Da, an average mass of 2688.14 Da, and a theoretical pI of 8.36. Trypsin digestion generated the peptide residues 409-431 (TTGYLYIPALAALHSPSSLLSPQ), with a monoisotopic mass of 2399.44 Da, an average mass of 2400.76 Da, and a theoretical pI of 6.40. The expected PEPMASS for the singly protonated species ([M+H]^+^) is 2449.34 Da, and for the doubly charged species ([M+2H]^2+^) is 1225.17 Da. Detailed tryptic peptide sequences and properties are provided in Table S1.

#### FANTOM5 CAGE data processing

Strand-specific CAGE tag counts (CTSS) for exon 12 of *FGGY*-204 (ENST00000371218) were retrieved from FANTOM5 HepG2 (three biological replicates) and liver (two pooled samples: adult and fetal) datasets. BED files were imported as GRanges objects with 1 kb padding around the exon. Strand-specific signals were summed per sample, and a bidirectionality ratio was computed as the ratio of the smaller to larger strand signal.

#### Whole-exome sequencing, tumor mutational burden, and microsatellite instability

Whole-exome sequencing (WES) data used in this study were obtained from the National Translational Science Network of Precision-Based Immunotherapy for Primary Liver Cancer and are available through the database of Genotypes and Phenotypes (dbGaP) under accession phs003074.v1.p1. Due to patient privacy and controlled-access requirements, these data are not publicly available. Researchers may request access through dbGaP by submitting a data access request via the NIH Data Access Committee, which oversees and regulates data use. Details on how to apply for access, including submission procedures and data use limitations, are available via the dbGaP study page for accession phs003074.v1.p1.

Tumor and matched normal samples were processed using standard WES pipelines. Briefly, raw reads were trimmed with Trimmomatic,^42^ aligned to the human reference genome using BWA-MEM,^43^^,^^44^ and duplicates were marked with Picard. Variants were called with GATK HaplotypeCaller for germline variants and Mutect2 for somatic variants. Somatic variants were filtered for high-confidence PASS calls and annotated with Funcotator. Tumor mutational burden (TMB; mutations per megabase) was quantified using pyTMB^45^ on tumor-only somatic VCFs, reporting Total TMB, Clonal TMB (VAF ≥0.3), and Subclonal TMB (VAF <0.3). Microsatellite instability (MSI) was assessed using MANTIS^46^ via the DIF score and classified as MSI-High (DIF >0.35), MSI-Low (0.20-0.35), or MSS (≤0.20). FGGY alteration status (missense, truncating/frameshift, splice) was derived from normalized, annotated calls. Nonsynonymous and frameshift FGGY variants were translated into mutant peptides to evaluate HLA class I binding (NetMHCpan), with predicted IC50 < 500 nM considered candidate neoantigens. Samples were clustered into Hot, Intermediate, and Cold groups based on transcriptional immune signatures, immune cell infiltration scores, TMB, and MSI profiles using hierarchical clustering (Ward’s method). Associations between FGGY status, MSI, TMB, and predicted neoantigens were evaluated using non-parametric tests and Spearman correlations with multiple-testing correction.

#### Assessment of immune cell infiltration and tumor microenvironment composition

To evaluate immune cell infiltration and the tumor microenvironment from RNA-seq data, multiple computational deconvolution approaches were employed. The Tracking Tumor Immuno-Phenotype (TIP) algorithm quantified anti-tumor immune activity across the cancer-immunity cycle.^47^ In parallel, xCell^48^ estimated relative abundances of various immune and stromal cell types. Gene expression values were normalized and scaled prior to analysis. Cluster-specific immune profiles were derived by integrating these scores with transcriptional signatures, enabling classification of samples into hot, intermediate, and cold immune phenotypes.

#### Proteomic validation

Public datasets (including PDC000219, PDC000233, PDC000234, PDC000153) were analyzed for peptides derived from the FGGY-204-specific exon 12 sequence. Peptide-spectrum matches (PSMs) were filtered for high confidence (rank 1, FDR <1%). A 27-amino-acid peptide (TTGYLYIPALAALHSPSSLLSPQVTGLK) spanning the exon 12-encoded region was identified with multiple high-confidence spectra across several cancer types, including lung adenocarcinoma (LUAD), lung squamous cell carcinoma (LSCC), breast cancer, colon cancer, head and neck cancer (HNSCC), clear cell renal cell carcinoma (CCRCC), and glioblastoma (GBM), confirming the translation of the FGGY-204 isoform.

The detected peptides collectively cover a large portion of the FGGY-204 unique sequence and can be categorized into three overlapping groups: A long, core 61-amino-acid peptide (AQPVGFLTVDLHVWPDFHGNRSPLADLTLKGMRTTGYLYIPALAALHSPSSLLSPQVTGLK) was frequently identified. This peptide spans the junction upstream of exon 12 and contains the entire 25-amino-acid sequence, including the core immunogenic epitope YLYIPALAA. Shorter, specific peptides corresponding to the N-terminal region (KAQPVGFLTVDLHVWPDFHGNRSPLADLTLKGMR), the central exon 12-containing region (TTGYLYIPALAALHSPSSLLSPQVTGLK), and the C-terminal region (SPLADLTLKGMRTTGYLYIPALAALHSPSSLLSPQVTGLKLSQDLDDLAILYLATVQAIALGTR) were also detected, providing redundant validation. Very long peptides (>70 AA) covering almost the entire unique FGGY-204 sequence were identified, further confirming its translation. Notably, the core 61-AA peptide was found to be extensively post-translationally modified, with multiple high-confidence phosphorylation sites identified on serine within the exon 12-encoded sequence (serine sites: S18, S20, S21, S24, S33). This suggests the FGGY-204 isoform is not only translated but also functionally regulated in cancer cells. In conclusion, this comprehensive proteomic analysis provides robust evidence for: the translation of the FGGY-204-specific isoform into protein; its expression across a wide range of human cancers, and its regulation via phosphorylation, potentially modulating its function or immunogenicity.

#### IEDB analysis

IEDB analysis resource identified four human peptides with >70% sequence similarity to the FGGY-204 core epitope, derived from SH2 domain-containing adapter protein B (*SHB*; VTIPALAAQF), Importin-4 (*IPO4*; GEAIPALAA), Guanine nucleotide-binding protein subunit alpha-12 (*GNA12*; LYVPALSALW), and Olfactory receptor 52R1 (*OR52R1*; KAFSTRSSHICVILALYIPALF). None of these peptides were predicted as strong binders for either MHC class I or class II alleles. Weak binding was observed for IPALAAQF under HLA-B∗*07:02* and for LYVPALSAL under HLA-C*∗*07:02. Several OR52R1-derived peptides also showed weak binding signals across multiple alleles, including HLA-A∗02:01 (VILALYIPA, VILALYIPAL, ILALYIPA, ILALYIPALF), HLA-A∗03:01 (HICVILALY), HLA-B∗08:01 (ILALYIPAL), and HLA-C∗07:02 (TRSSHICVI, SHICVILAL, TRSSHICVIL). Critically, none of these similar peptides were predicted as strong binders for either MHC class I or class II alleles, minimizing potential cross-reactivity concerns.

#### Exonic splicing enhancer analysis

To illustrate the regulation of exon 12 inclusion in *FGGY*, we examined splicing regulatory elements and the chromatin context at the gene locus. Using *ESEfinder*,^23^ we identified multiple strong exonic splicing enhancer motifs within exon 12, that are specifically recognized by serine-arginine rich RNA splicing (SR) proteins, including SRSF1, SRSF2, SRSF5, and SRSF6 (Figure S2F). All motifs scored above established thresholds, suggesting a strong RNA splicing activity at exon 12.

#### RNA-binding protein (RBP) and transcription factor (TF) analysis

RBP predictions for exon 12 inclusion: To investigate trans-acting factors that may contribute to context-specific inclusion of exon 12 in FGGY-204, we predicted RBP binding to the exon 12 sequence using RBPmap. High-confidence candidates (A1CF, KHDRBS3, SRSF10, PUF60, U2AF2, PTBP3, TIA1, and HNRNPC) were identified based on a weighted ranking. Across intron 11 and exon 12, 126 RBPs were predicted to bind after applying conservation and statistical filters (Z-score ≥ 2, P-value <0.05, Table S2). Expression-based filtering (|log2FoldChange| > 0.01, P-value(adj) < 0.05, baseMean >5) comparing HCC tumor (HCC.T) and non-tumor (HCC.NT) samples retained 26 RBPs. Among these, 12 RBPs are functionally associated with 3′-splice site selection, intron retention, and exon inclusion/skipping, explaining up to 92% of the variance in FGGY isoform expression. Variance of the FGGY-204 isoform is predominantly accounted for by PUF60, HNRNPA1, and PCBP2. TF binding predictions Promoter (0.7 kb upstream) and putative enhancer regions of FGGY were scanned for TF binding sites using ENCODE and motif databases. Predicted TFs in the promoter region include KLF6, SP1, YY1, CTCF, HNF4A, and MYC (FGGY.TF.0.7kb), whereas enhancer regions contain predicted binding sites for KLF6, SP1, YY1, FOXA1, EP300, SMARCA4, and POLR2A (FGGY.TF.0.enhancer).

#### DNA methylation and ChIP-seq data analysis

To further understand the epigenetic regulation of FGGY, we assessed DNA methylation patterns and repressor complex occupancy at this locus. Whole-genome bisulfite sequencing (WGBS) data from liver tissue revealed consistently high levels of DNA methylation across the *FGGY* gene body, with relatively lower methylation near the promoter. This pattern of gene-body methylation is often associated with actively transcribed genes, but in certain contexts, such as those marked by poised chromatin states, it may reflect transcriptional suppression. Indeed, reference data from HepG2 histone profiling suggests a poised or bivalent chromatin configuration at the *FGGY* promoter. Notably, methylation levels differed significantly between tumor and non-tumoral samples, with tumors exhibiting elevated methylation, particularly within exonic and intronic regions. To assess potential transcriptional repressors, we analyzed ChIP-seq data from 208 transcription factors in HepG2 cells. Most transcription factors displayed no more than two binding peaks within the *FGGY* locus (data not shown). However, the COREST complex (GSM935579) was a striking exception, with 13 distinct peaks distributed throughout the gene body (three located within exons, one peak at the promoter, and the remainder in introns. COREST is a component of the BHC (BRAF–HDAC) complex, which facilitates transcriptional silencing through recruitment of histone demethylases such as KDM1A and methyl-CpG-binding proteins like MeCP2. The co-localization of COREST binding and regions of high DNA methylation suggests a cooperative repressive mechanism that may regulate *FGGY* transcription or isoform usage. MeCP2 recruits the CoREST complex to methylated DNA regions, and its knockout in iWAT and BAT leads to decreased FGGY expression in iWAT and increased expression in BAT. This suggests that MeCP2 and CoREST may have cell- or tissue-specific regulatory roles, influencing chromatin states and metabolic gene expression programs.

#### Transcription and splicing factors analysis

To identify potential regulators of *FGGY* isoform expression, we performed a correlation analysis between the expression of all annotated human transcription factors (TFs) and splicing factors (SFs) and the three major *FGGY* isoforms (-201, -202, -204) from RNA-seq data. We defined two distinct regulatory modules based on opposing Spearman correlation patterns: Module T1 consisted of 24 genes that exhibited significant and opposite correlations (|ρ| > 0.5, p < 0.05) with the FGGY-201 and FGGY-202 isoforms. Module T2 consisted of 12 genes that showed significant and opposite correlations (|ρ| > 0.4, p < 0.05) with the FGGY-201 and FGGY-204 isoforms. Transcription factor enrichment analysis on the gene modules was performed using the ChEA3 tool. This analysis revealed that eight genes within Module T2 (ESRRA, REPIN1, ZNF628, ZNF219, RP9, ZNF865, E4F1, ZBTB7B) were significantly enriched for targets of ZNF784. The correlation of key genes from Module T1 (including KDM8, ZNF414, MXD3, SETDB1, and SMARCA4) with *FGGY* isoforms was validated in an independent cohort (GSE77314), where they maintained a significant correlation (|ρ| > 0.5, p < 0.05) with *FGGY-201*.

#### Transcription start site and transcriptional regulator analysis

##### Promoter and transcription factor (TF) binding site analysis

The regulatory architecture of the *FGGY* promoter was characterized by analyzing the region 700 bp upstream and downstream of its primary Transcription Start Site (TSS), as annotated in public databases (GeneCards). Putative TF binding sites within this region were identified from the same source.

##### Differential TF expression and correlation

To identify TFs with potential regulatory roles in HCC, their expression was compared between HCC tumor (HCC.T) and non-tumoral (HCC.NT) tissues. The correlation between the expression levels of significantly dysregulated TFs and the abundance of specific *FGGY* isoforms was then calculated. This analysis identified SMARCA4, ZNF205, MXD3, and ZNF580 as candidates with significant correlations to *FGGY* isoform abundance (Figures S4A and S4F).

##### Co-expression network analysis

A Weighted Gene Co-expression Network Analysis (WGCNA) was performed to place *FGGY* within a functional regulatory context. This identified a module of co-expressed genes, which was subsequently analyzed for functional enrichment using Gene Ontology (GO) terms.

#### Untargeted metabolomics analysis

FGGY catalyzes the ATP-dependent phosphorylation of D-ribulose, contributing to metabolite repair and the recycling of ribulose-5-phosphate in the PPP. Its zonal enrichment in periportal hepatocytes aligns with zone 1 metabolic programs, including oxidative metabolism and detoxification. Untargeted metabolomics was performed as described in our previous works.^30^ Metabolomic profiling was performed using LC–MS on a Sciex TripleTOF 6600 system coupled to an Agilent 1290 HPLC platform, with separate workflows for polar metabolites and lipids using HILIC and reverse-phase (RP) chromatography, respectively. Data were acquired in both positive and negative ionization modes. Data-dependent acquisition (DDA) was conducted on pooled quality control (QC) samples for compound identification, while SWATH-MS (data-independent acquisition, DIA) was applied to individual samples for relative quantification. Raw data files were converted to mzML format and processed using MetaboKit (Narayanaswamy et al., 2020). To maximize metabolite coverage, we implemented a curated pipeline integrating both MS/MS-based identifications (MS2 level; n = 915) and accurate mass-based annotations (MS1 level; n = 548). A project-specific spectral library generated from DDA data was used to extract MS1 and MS2 features from SWATH acquisitions via the MetaboKit DIA module. Quality control filtering was applied to ensure robust quantification. Compound transitions with a coefficient of variation (CoV) > 30% across QC samples were excluded. For each metabolite, the precursor or fragment ion exhibiting the lowest CoV was selected as the quantifier. When metabolites were detected in both positive and negative ionization modes, or across both RP and HILIC platforms, the feature with the lowest CoV was retained. Overlapping detections between RP and HILIC primarily involved semi-polar lipids; in such cases, measurements from the RP method were prioritized. Samples were analyzed in randomized order within each batch, with pooled QC samples injected every ten runs to monitor instrument stability and reproducibility. Free ribulose and ribitol were not detected, consistent with prior studies where their accumulation required specific genetic and nutritional perturbations.^20^ For the FGGY-201 Association: “The specific association of the catalytic FGGY-201 isoform, but not other isoforms, with α-D-glucose and D-ribose 5-phosphate suggests a potential role for this isoform in glucose sensing or metabolic crosstalk between glycolysis and the PPP.

#### Immune landscape analysis

##### Cohort

The NCI-CLARITY ICI-treated HCC cohort (dbGaP: phs003074.v1.p1) was used for all longitudinal immune analyses.

##### Immune classification

Tumor immune status was assessed using a published 18-gene IFN-γ-responsive signature (Ayers et al., JCI 2017). Ward’s hierarchical clustering was applied to this signature to classify tumors into “hot,” “intermediate,” and “cold” groups.

##### Immune activity quantification

The Tumor Immune Profiling (TIP) algorithm was used to score the activity of each step in the cancer-immunity cycle. Statistical significance across timepoints was assessed using the Kruskal-Wallis test, with post-hoc Dunn’s test for pairwise comparisons. A mixed-effects model was used to confirm the effect of timepoint while accounting for patient-level variation.

##### Immune cell deconvolution and correlation

Immune cell compositions were estimated from bulk RNA-seq data using CIBERSORTx with the LM22 signature matrix. Analyses were performed separately on the MASLD-HCC cohort and the NCI-CLARITY cohort (ICI-treated). Correlations between *FGGY* expression and immune cell fractions were calculated using Spearman’s rank correlation (ρ). P-values were adjusted for multiple testing using the Benjamini-Hochberg method where indicated.

##### Mixed-effects modeling of gene expression

Gene-level mixed-effects modeling was performed on the NCI-CLARITY cohort to identify genes with significant temporal expression shifts. The model treated patient as a random effect and timepoint as a fixed effect. Genes with a significant timepoint effect (FDR <0.05) were considered dynamically regulated.

#### Somatic variant analysis

##### Variant calling

Coding somatic variants in the *FGGY* gene were identified from whole-exome sequencing data using a GATK/Mutect2 pipeline.

##### Filtering & annotation

All reported variants passed Mutect2 quality filters. They were annotated against gnomAD and clinical databases. Variant allele frequency (VAF) was used to infer clonality (VAF >0.3 suggesting clonal).

##### Identified variants

p.A290V (rs998421142): Reported in clinical variants as p.A290T; VAF = 0.10 (Subclonal).

p.G105W (rs1394238610): Reported in gnomAD as p.G105E; VAF = 0.87 (Clonal).

p.V191M (rs72913795): VAF = 0.85 (Clonal).

p.G330D (rs781638004): Reported in gnomAD as p.G330A; VAF = 0.25 (Subclonal).

### Quantification and statistical analysis

All statistical analyses were performed using R (version 4.4.2) and associated packages unless otherwise specified. Details of specific statistical tests, sample sizes, and significance thresholds are provided in the figure legends and results section.

For transcriptomic analyses, differential expression and isoform usage were assessed using established pipelines, including DEXSeq, with statistical significance defined by a false discovery rate (FDR) ≤ 0.05 unless otherwise stated.

Correlation analyses were performed using Spearman’s rank correlation coefficient (ρ), with p values adjusted for multiple testing using the Benjamini-Hochberg method where applicable. Non-parametric statistical tests, including the Kruskal-Wallis test followed by Dunn’s post hoc test, were used for comparisons across multiple groups. Pairwise comparisons were performed using Wilcoxon rank-sum tests unless otherwise specified.

Tumor mutational burden and microsatellite instability classifications were derived using established thresholds as described in the method details section. Associations between genomic features and immune or transcriptional variables were evaluated using non-parametric tests and correlation analyses.

For immune deconvolution analyses, statistical comparisons and correlations were performed on normalized gene expression data. Multiple-testing correction was applied where appropriate.

Sample size (n) represents the number of biologically independent samples unless otherwise indicated. For analyses involving public datasets, n corresponds to the number of samples available within each dataset after quality control filtering.

Data are presented as mean ± standard deviation (SD), as indicated in the figure legends. A significance threshold of p < 0.05 was used unless otherwise specified.

## Supplemental information

Document S1. Figures S1–S7

Table S1. Core epitope identification, IEDB binding predictions, proteomic validation, and IEDB analysis, related to STAR Methods

Table S2. High-confidence RBPs predicted to regulate exon 12 splicing, related to STAR Methods

Table S3. RNA-seq and metabolomic data of MASLD-associated HCC, related to STAR Methods
