# Supplementary material for: Alternative splicing regulates FGGY-derived neoantigen presentation and promotes immune evasion in metabolic-associated hepatocellular carcinoma
Source: iScience. 2026 Jun 6;29(6):115999. doi: 10.1016/j.isci.2026.115999 (PMC13264262; doi:10.1016/j.isci.2026.115999)
Supplement: Document S1. Figures S1–S7 [file mmc1.pdf]

iScience, Volume *29*

Supplemental information

Alternative splicing regulates *FGGY*-derived neoantigen presentation and promotes immune evasion in metabolic-associated hepatocellular carcinoma

Li Na Zhao and Jesper B. Andersen

Supplemental Information

Figure S1

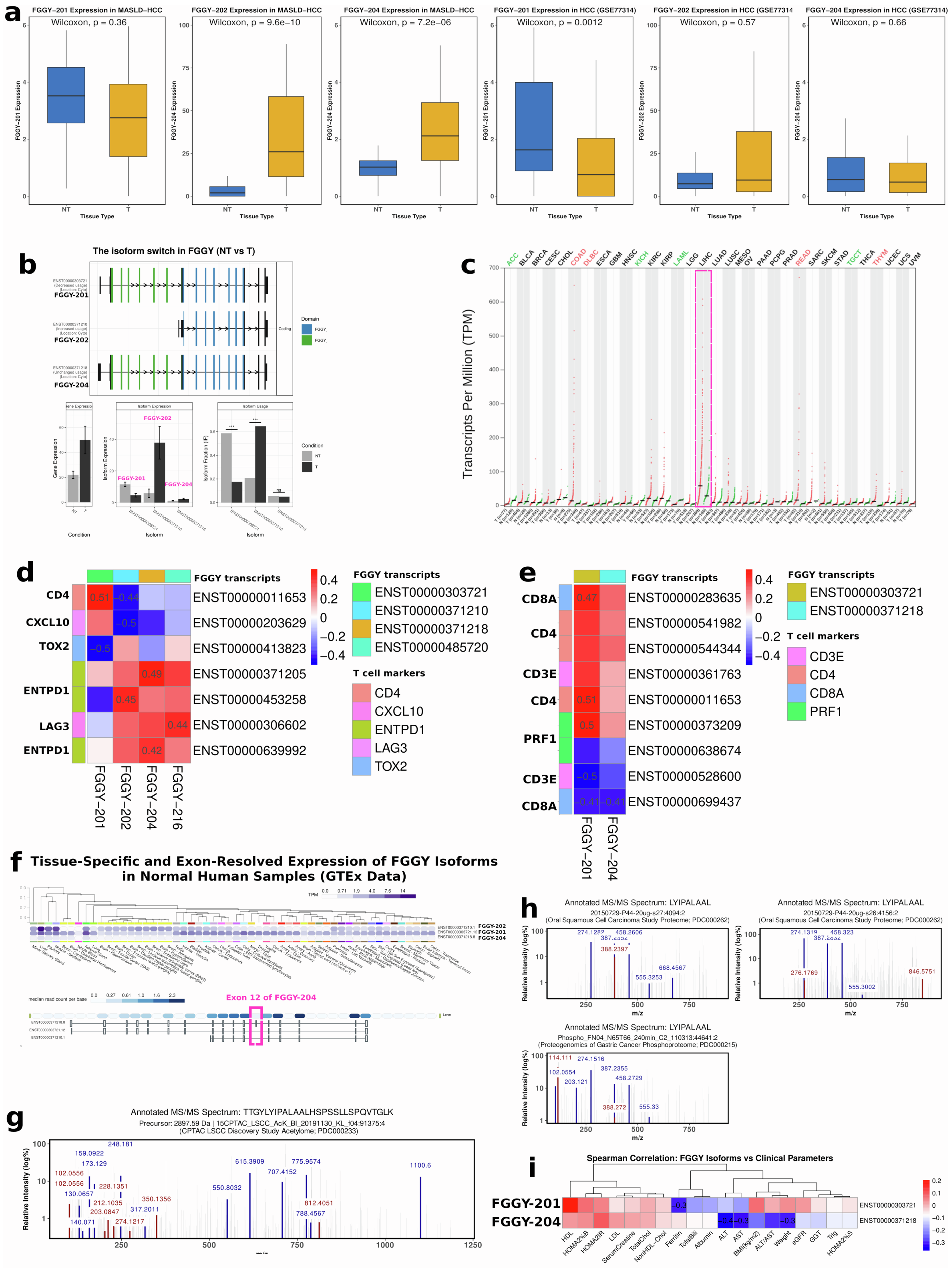

Figure S1. Isoform-specific expression and immune associations of FGGY in liver cancer and normal tissues. Related to Figure 1.

(a) Expression of FGGY-204 and FGGY-202 isoforms across MASLD-HCC samples and validated in HCC (GSE77314). Expression values are shown as boxplots; statistical comparisons were performed using the Wilcoxon test.

(b) Isoform switch plot of FGGY comparing HCC adjacent non-tumor (HCC.NT) and tumor (HCC.T) samples in the MASLD-HCC dataset.

(c) FGGY expression profile across tumor and paired normal samples from multiple cancer types (data from GEPIA: Gene Expression Profiling Interactive Analysis).

(d) Correlation between T-cell markers and FGGY isoforms in MASLD-HCC. Heatmap displays Pearson correlation coefficients between FGGY isoform expression and T-cell marker genes. Red indicates positive correlation, blue indicates negative, with color intensity reflecting correlation strength.

(e) Correlation between T-cell markers and FGGY isoforms in GSE77314.

(f) FGGY isoform expression in normal human tissues based on GTEx. The upper panel shows isoform-level expression across a wide range of tissues, highlighting tissue-specific distribution. The middle panel shows exon-level expression of FGGY in normal liver, highlighting minimal usage of exon 12 specific to FGGY-204.

(g) Tandem mass spectrometry (MS/MS) analysis of the FGGY-204 exon 12: TTGYLYIPALAALHSPSSLLSPQVTGLK.

(h) Tandem mass spectrometry (MS/MS) analysis of the FGGY-derived immunogenetics peptide LYIPALAAL. Annotated MS/MS spectra from four independent proteomic datasets show fragmentation patterns consistent with the core epitope sequence. Top: Spectra from Oral Squamous Cell Carcinoma Study Proteome (PDC000262) demonstrating characteristic b- and y-ion series. Bottom: Spectra from Gastric Cancer Phosphoproteome (PDC000215). Red labels indicate b-ions (N-terminal fragments), blue labels indicate y-ions (C-terminal fragments).

(i) Heatmap showing Spearman correlations between FGGY isoforms and clinical parameters. Color intensity reflects correlation strength (blue: negative, red: positive).

Figure S2

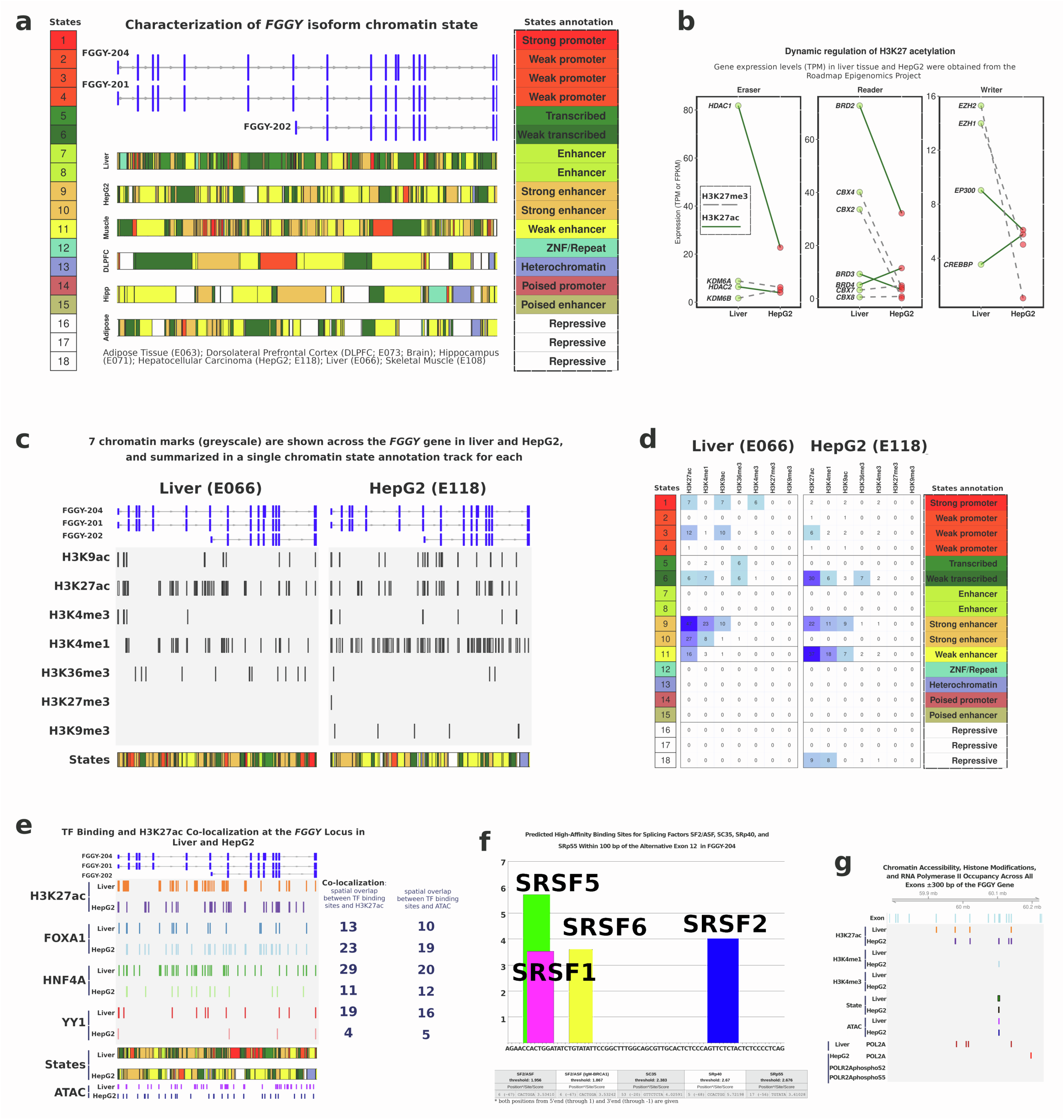

Figure S2. Epigenomic regulation of the FGGY locus reveals tissue-specific chromatin states and transcription factor occupancy. Related to Figure 2.

(a) Chromatin state landscape at the FGGY locus (TSS + 2 kb) across five tissues. Gene region tracks and ChromHMM-derived chromatin state annotations are shown for Liver (E066), HepG2 (E118), Brain (E071: Hippocampus; E073: DLPFC), Muscle (E108), and Adipose (E063). Active promoter and

enhancer states are most enriched in liver, while HepG2 displays increased weak, poised, or repressed states. Genomic coordinates are based on the hg19 reference genome.

(b) Dynamic expression of H3K27 histone modification regulators in liver and HepG2. Expression levels of key H3K27 acetylation and methylation writers, erasers, and readers are compared between the two samples. Writers and readers of H3K27ac (e.g., EP300, BRD2/3/4) show distinct expression patterns from methylation-associated factors (EZH2, CBX family). Notably, several acetylation erasers (HDAC1/2) and readers exhibit altered expression in HepG2, consistent with epigenetic remodeling in the cancer context.

(c) Profiles of seven chromatin marks (greyscale) across the FGGY gene in liver and HepG2. Individual ChIP-seq signal tracks for each histone mark are shown, with an integrated ChromHMM-derived chromatin state annotation track below, color-coded as in (A). This highlights cell type-specific chromatin landscape differences at the FGGY locus.

(d) Heatmap of histone mark enrichment at the FGGY locus in liver and HepG2.

(e) Integrated epigenomic landscape and co-occupancy at the FGGY locus. Left: Tracks show gene structure, histone modification ChIP-seq, FOXA1, HNF4A, and YY1 ChIP-seq, ChromHMM chromatin states, and ATAC-seq accessibility in liver and HepG2. Right: Overlap of TF binding sites with both H3K27ac peaks and ATAC-seq accessible regions highlights candidate regulatory elements.

(f) Predicted SRSF binding motifs around exon 12 of FGGY. ESEfinder analysis identified putative exonic splicing enhancer (ESE) motifs for SR proteins (SRSF1, SRSF2, SRSF5, and SRSF6) within exon 12 of the FGGY gene. These predicted binding sites suggest potential regulatory roles for these SR proteins in promoting exon 12 inclusion via recognition of ESE motifs.

(g) Integrated genomic tracks displaying FGGY exons (±300 bp), histone modifications, chromatin states, ATAC-seq accessibility, and POLR2A binding profiles in liver and HepG2 cells within the specified genomic region.

Figure S3

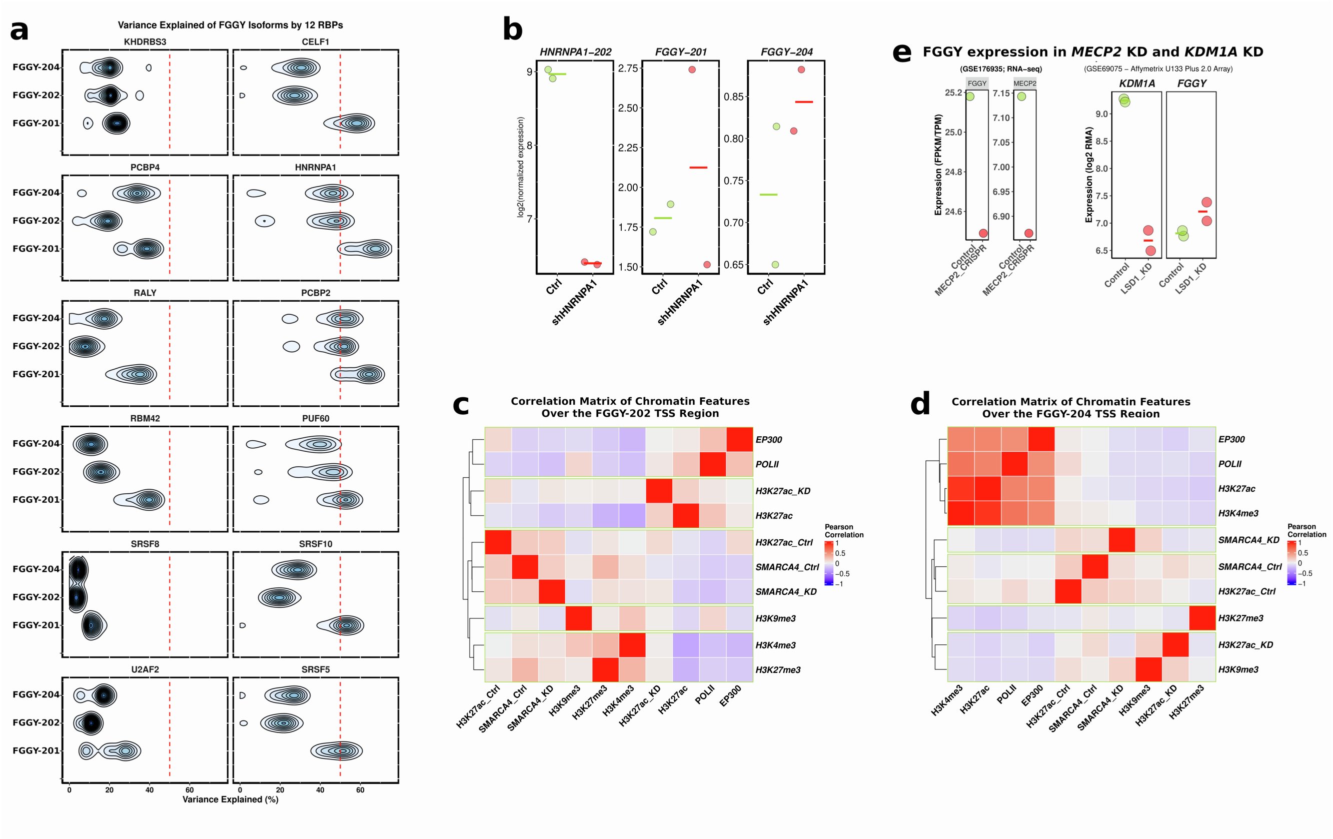

Figure S3. Isoform-specific regulation and chromatin landscapes of FGGY. Related to Figure 2 and 3.

(a) Variance explained in expression of FGGY isoforms by individual RNA-binding proteins (RBPs). Each facet shows the distribution of variance explained (%) for the three major FGGY isoforms (FGGY-201, FGGY-202, FGGY-204) when modeled using the expression of a single RBP transcripts expression level. The variance was computed using partial least squares regression across 10-fold cross-validation (10 repeats). Density plots represent the spread of explained variance across cross-validation folds, with dashed red lines indicating high-variance thresholds.

(b) Expression levels of FGGY and HNRNPA1-202 isoforms following HNRNPA1 knockdown. Transcript-level expression (log_2TPM) of three FGGY isoforms (FGGY-201, -204) and the HNRNPA1-202 isoform (ENST00000340913) in control and HNRNPA1 knockdown conditions. Control samples correspond to ENCODE files ENCFF942RUQ and ENCFF977QOX. Knockdown samples were generated using two independent shRNAs: ENCFF289RGO and ENCFF946WES. Each point represents a sample replicate. Effective reduction of HNRNPA1-202 expression validates knockdown, with associated changes in FGGY isoform expression suggesting potential isoform-level regulation by HNRNPA1.

(c-d) Isoform-specific chromatin landscapes at the FGGY-202 and FGGY-204 locus. Heatmaps of Pearson correlations among ChIP-seq signals for active (H3K9ac, H3K27ac, H3K4me3, POLII,

EP300) and repressive (H3K27me3, H3K9me3) marks at the ±5 kb region surrounding the transcription start site (TSS) of FGGY-202, and FGGY-204.FGGY-204 promoters show strong correlations among active marks, indicative of transcriptionally engaged chromatin, which is partially disrupted upon SMARCA4 knockdown. FGGY-202 displays weaker correlations among active marks, suggesting lower chromatin engagement and reduced dependence on SWI/SNF-mediated remodeling. Repressive marks show weak or negative correlations with active marks across both isoforms, reflecting mutually exclusive chromatin states. The ChIP-seq data of HepG2 cells are from ENCODE.

(e) Isoform-specific expression of FGGY in MECP2 and KDM1A knockdowns.

Figure S4

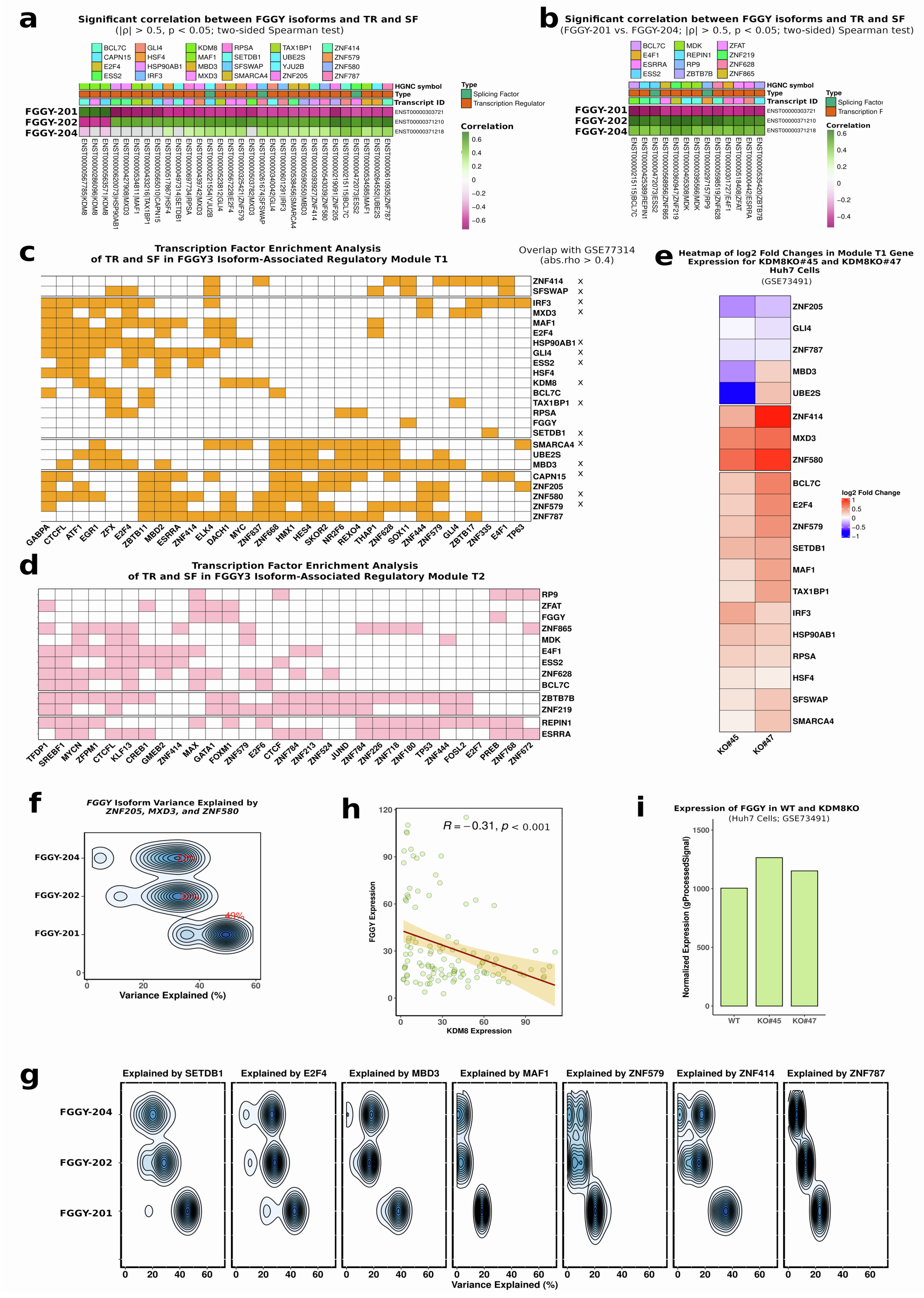

a
Significant correlation between FGGY isoforms and TR and SF
(|ρ| > 0.5, p < 0.05; two-sided Spearman test)
FGGY-201
FGGY-202
FGGY-204
HGNC symbol
Type
Transcript ID
Splicing Factor
Transcription Regulator
Correlation
b
Significant correlation between FGGY isoforms and TR and SF
(FGGY-201 vs. FGGY-204; |ρ| > 0.5, p < 0.05; two-sided) Spearman test)
c
Transcription Factor Enrichment Analysis
of TR and SF in FGGY3 Isoform-Associated Regulatory Module T1
Overlap with GSE77314
(abs.rho > 0.4)
d
Transcription Factor Enrichment Analysis
of TR and SF in FGGY3 Isoform-Associated Regulatory Module T2
e
Heatmap of log2 Fold Changes in Module T1 Gene
Expression for KDM8KO#45 and KDM8KO#47
Huh7 Cells
(GSE73491)
log2 Fold Change
KO#45
KO#47
f
FGGY Isoform Variance Explained by
ZNF205, MXD3, and ZNF580
Variance Explained (%)
h
R = −0.31, p < 0.001
FGGY Expression
KDM8 Expression
i
Expression of FGGY in WT and KDM8KO
(Huh7 Cells; GSE73491)
Normalized Expression (gProcessedSignal)
WT
KO#45
KO#47
g
Explained by SETDB1
Explained by E2F4
Explained by MBD3
Explained by MAF1
Explained by ZNF579
Explained by ZNF414
Explained by ZNF787
Variance Explained (%)

Figure S4. Integrated regulatory landscape of FGGY isoforms reveals transcriptional and splicing control. Related to Figure 4.

(a) Heatmap showing significant correlations ($|\rho| > 0.5$; $p<0.05$; Speareman) between FGGY isoforms and regulatory genes (Module T1), including ranscription Regulators (TR) and Splicing Factors (SF). Correlation coefficients (Spearman's ρ) between isoform expression and regulator isoforms are color-coded, with annotations indicating regulator type. Clustering highlights patterns of co-expression relevant to FGYY different isoforms.

(b) Heatmap showing significant correlations between FGGY-201 and FGGY-204 and regulatory genes.

(c) Transcription factor enrichment analysis for Module T1.

(d) Transcription factor enrichment analysis for Module T2.

(e) Heatmap displays the log2 fold changes of Module T1 genes in two knockout samples, KDM8KO#45 and KDM8KO#47, relative to wild type. The color gradient from blue to red represents downregulation to upregulation, respectively. Genes are clustered by similarity in expression changes across both knockout conditions, highlighting consistent and divergent transcriptional responses to the KDM8 knockout.

(f) Density distribution of variance explained by *ZNF205*, *MXD3*, and *ZNF580* for three FGGY isoforms.

(g) Density distribution of variance explained by transcription repressor expression for three FGGY isoforms.

(h) FGGY expression positively correlates with KDM8 expression across samples. Scatter plot showing the relationship between KDM8 and FGGY expression levels across samples. Spearman correlation coefficient and significance are indicated, supporting a transcriptional association between KDM8 and FGGY.

(i) FGGY expression is upregulated in JMJD5 (KDM8) knockout Huh7 cells. Bar plot showing normalized FGGY mRNA expression levels in wild-type (WT) and JMJD5 knockout (KO#45 and KO#47) Huh7 samples.

Figure S5

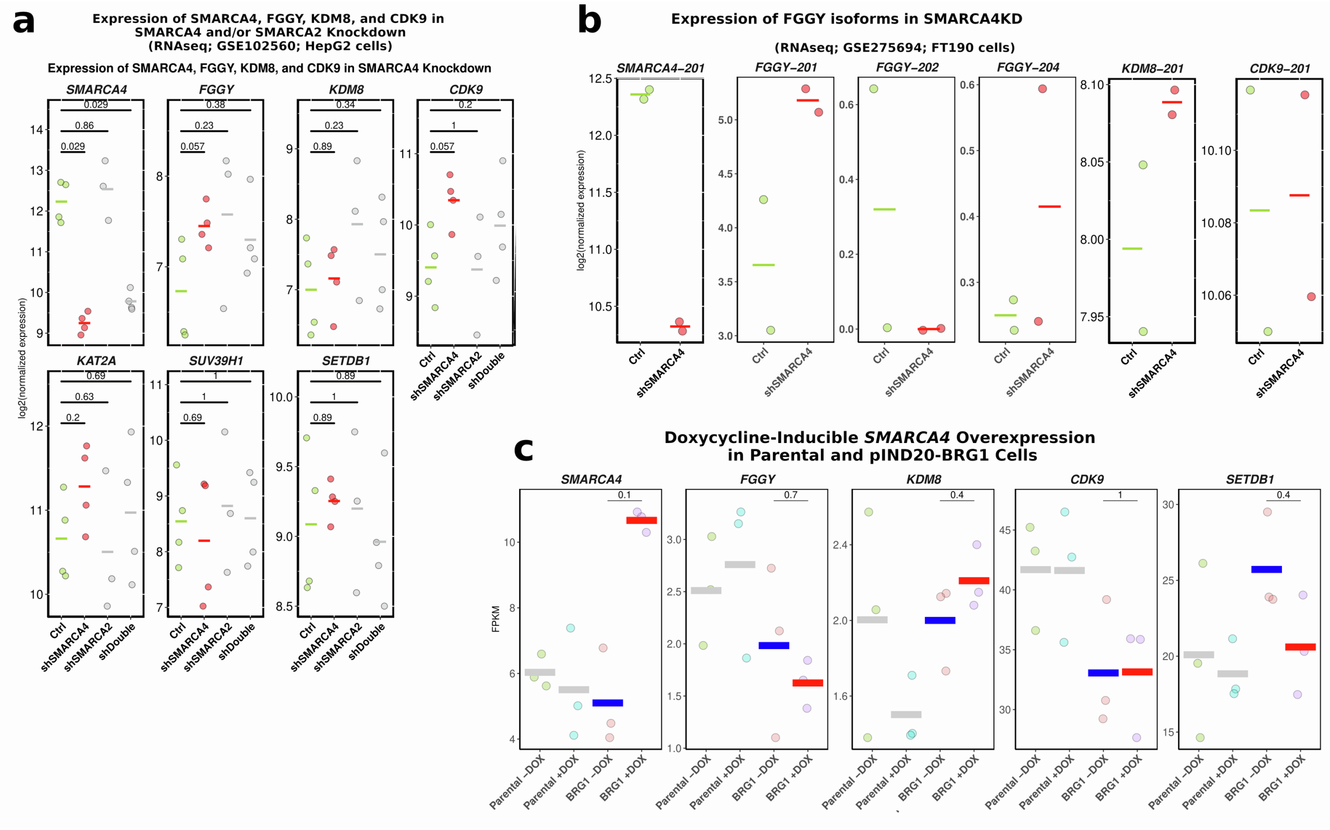

Figure S5. Regulation of FGGY and chromatin modifiers upon SMARCA perturbation. Related to Figure 4.

(a) Expression of key chromatin regulators and FGGY following SMARCA4 and/or SMARCA2 knockdown. Jitter plots display individual log2-normalized expression values for control (Ctrl), SMARCA4 knockdown (shSMARCA4), SMARCA2 knockdown (shSMARCA2), and double knockdown (shDouble) conditions, with crossbars representing mean expression. Pairwise Wilcoxon tests were used for statistical comparisons. Data from GSE102560 RNA-seq.

(b) Isoform-specific expression of FGGY in SMARCA4 knockdown cells.

(c) Expression of FGGY, KDM8, CDK9, and SETDB1 following SMARCA4 overexpression.

Figure S6

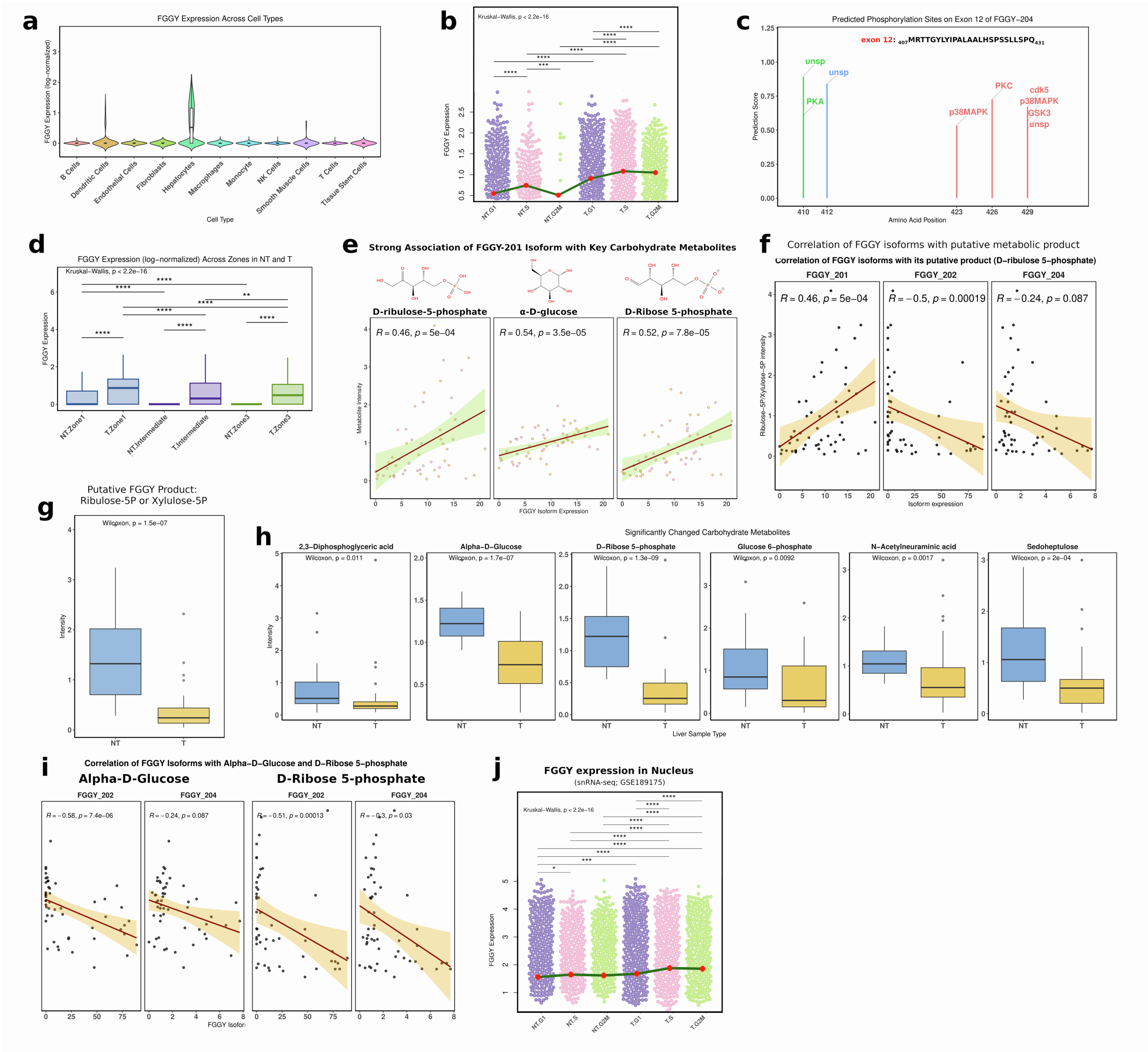

Figure S6. Isoform-, spatial-, and metabolite-associated regulation of FGGY in liver and HCC. Related to Figure 1 and STAR Methods.

(a) FGGY expression is enriched in hepatocytes. Violin and box plot showing the distribution of log-normalized FGGY expression across major liver cell types. Hepatocytes display the highest expression levels of FGGY compared to other cell types, indicating cell type-specific enrichment. Data are derived from scRNA-seq (GSE149614) of liver tissues.

(b) FGGY expression across cell cycle phases in hepatocytes from non-tumor and tumor liver tissue, shown separately for nuclear compartment. Beeswarm plots display individual log-normalized FGGY expression values grouped by cell cycle phase. Statistical comparisons between groups were conducted using Wilcoxon rank-sum tests for pairwise comparisons and the Kruskal-Wallis test for overall group

differences. Significance is denoted as follows: ns (not significant): $p > 0.05$ (not shown); *: $p \leq 0.05$; **: $p \leq 0.01$; ***: $p \leq 0.001$; ****: $p \leq 0.0001$. This significance notation applies to all other figures unless stated otherwise.

(c) Predicted phosphorylation sites on exon 12 of the FGGY-204 isoform. Isoform-specific regulation was explored using NetPhos v3.1, which identified multiple high-confidence serine (S) and threonine (T) residues in the FGGY-204-specific exon 12 sequence, predicted to be targets of kinases such as PKA, p38MAPK, PKC, CDK5, and GSK3. The x-axis shows amino acid positions, and the y-axis indicates the NetPhos prediction score. Label are the predicted kinase targets include PKA, p38MAPK, PKC, CDK5, and GSK3.

(d) FGGY expression across liver zonation categories in non-tumor and tumor hepatocytes. Log-normalized FGGY expression is shown for cells grouped by inferred zonation status (Zone 1, Intermediate, Zone 3) within each tissue type. Zonation categories were assigned based on relative module scores derived from established periportal (Zone 1: CPS1, HAL, ASS1) and pericentral (Zone 3: GLUL, CYP2E1, LGR5, AXIN2, TBX3) marker genes. Cells with a Zone1 module score at least 0.1 higher than Zone3 were labeled as Zone 1, and vice versa for Zone 3. All others were classified as Intermediate. Statistical comparisons were performed using Wilcoxon rank-sum tests.

(e) Correlation between FGGY-201 isoform expression and metabolite intensity. Spearman correlation analyses between the expression of the full-length FGGY-201 isoform and the intensities of three carbohydrate metabolites, D-ribulose 5-phosphate, α-D-glucose, and D-ribose 5-phosphate, identified from untargeted metabolomics. Scatter plots with linear regression lines and 95% confidence intervals are shown for each metabolite. FGGY-201 expression correlates strongly with D-ribulose 5-phosphate, a known enzymatic product of FGGY, and with two key intermediates of glycolysis and the pentose phosphate pathway.

(f) Correlation of FGGY isoforms with putative metabolic product.

(g) Decreased levels of putative FGGY products in tumor tissue. Boxplot showing the relative intensity of ribulose-5-phosphate or xylulose-5-phosphate, across liver sample types. Tumor tissues exhibit significantly lower levels compared to non-tumor samples, consistent with reduced expression of the full-length FGGY-201 isoform. Statistical comparisons were performed using the Wilcoxon test.

(h) Other carbohydrate metabolites showing significant differences between tumor and non-tumor samples.
(i) Correlations of FGGY-202 and FGGY-204 isoform expression with alpha-D-glucose and D-ribose-5-phosphate levels.

(j) FGGY isoform expression in the nucleus from single-nucleus RNA-seq (snRNA-seq; GSE189175), highlighting isoform-specific nuclear localization.

Figure S7

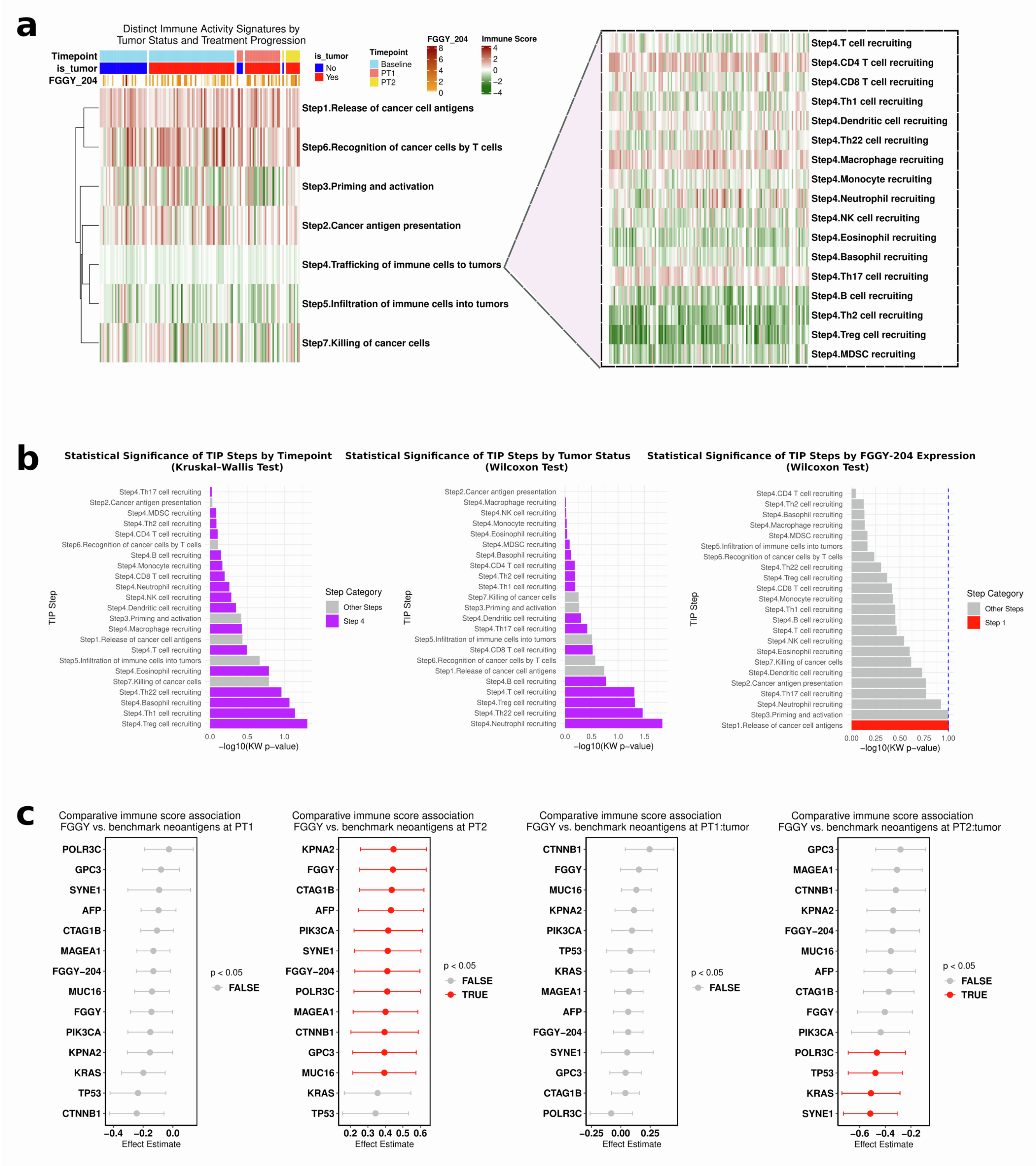

Figure S7. Immune landscape and gene expression associations across tumor status and treatment progression. Related to Figure 5.

(a) Heatmap of ssGSEA-derived immune scores showing distinct immune activity signatures across tumor status and treatment progression. Rows represent immunophenotyping; columns represent

individual samples grouped by tumor status and treatment stage. Color scale ranges from low (dark green) to high (dark red) immune activity. Top annotations indicate sample groupings, with rows clustered by similarity.

(b) Kruskal-Wallis test results for TIP steps across treatment groups. The left two panels highlight steps categorized as "Step 4" (purple) and "Step 1" (red), respectively, with steps ordered by ascending p-value (-log10 scale). The red panel includes a dashed blue line marking the significance threshold at $p = 0.1$.

(c) Effect estimates from linear mixed-effects models showing associations between gene expression and immune scores at PT1, PT2, and their interactions with tumor status, comparing FGGY-204 to benchmark immune-related genes. Significant effects ($FDR < 0.05$) are highlighted in red.
